# Meditation-Induced States, Vagal Tone, and Breathing Activity Are Related to Changes in Auditory Temporal Integration

**DOI:** 10.3390/bs9050051

**Published:** 2019-05-07

**Authors:** Damisela Linares Gutierrez, Sebastian Kübel, Anne Giersch, Stefan Schmidt, Karin Meissner, Marc Wittmann

**Affiliations:** 1 Institute for Frontier Areas of Psychology and Mental Health, 79098 Freiburg, Germany; linares@igpp.de (D.L.G.); sebastian.kuebel@web.de (S.K.); 2 INSERM U1114, 67091 Strasbourg, France; giersch@unistra.fr; 3 FMTS, Psychiatry Department, University Hospital of Strasbourg, 67200 Strasbourg, France; 4 Department of Psychosomatic Medicine and Psychotherapy, Medical Faculty, Medical Center—University of Freiburg, 79104 Freiburg, Germany; stefan.schmidt@uniklinik-freiburg.de; 5 Division of Integrative Health Promotion, Department of Social Work and Health, University of Applied Sciences, 96450 Coburg, Germany; karin.meissner@hs-coburg.de; 6 Institute of Medical Psychology, Ludwig-Maximilian University Munich, 80336 Munich, Germany

**Keywords:** mindfulness meditation, heart-rate variability, breathing rate, auditory temporal integration, present moment

## Abstract

This study is based on the relationship between meditation, the present moment, and psychophysiology. We employed the metronome task to operationalize the extension of the present moment. A pre-post longitudinal study was conducted. The performance in the metronome task was compared before and after the interventions (meditation, story). The aim was to assess whether physiological changes (heart, breathing) during meditation influence the temporal-integration (TI) of metronome beats. Mindfulness meditators either meditated (*n* = 41) or listened to a story (*n* = 43). The heart and breathing activity were recorded during the intervention and compared to a resting-state condition. By applying path analyses we found that meditation led to an increase of the duration of integration intervals at the slowest metronome frequency (inter-stimulus interval, ISI = 3 s). After meditation, the higher the heart-rate variability (i.e., the root mean square of successive differences, RMSSD), the longer the duration of integration intervals at the fastest frequency (ISI = 0.33 s). Moreover, the higher the breathing rate during meditation, the greater the integration of intervals at ISI = 1 s. These findings add evidence to meditation-induced changes on the TI of metronome beats and the concept of the embodiment of mental functioning.

## 1. Introduction

Recent conceptualizations emphasize the relationship between bodily processes, emotions, and time perception [1,2,3,4,5]. For example, the insula model of conscious awareness developed by Craig (2009) suggests that the perception of time is an embodied process in which the integration of signals from the body, as well as the awareness of related emotions, function as a subjective measure of time [1]. According to this theory, the insular cortex, along with other regions, plays a central role in the integration of interoceptive information in the brain which underlies the perception of the passage of time and temporal ordering [1,6].

Interoceptive abilities and the regulation of emotions are systematically fostered in certain meditative practices. Under attentive meditation instructions, for instance, while practicing the so-called body-scan, which is an integral part of the mindfulness-based, stress-reduction (MBSR) program [7], participants are told to strongly focus their attention on the sensations emanating from different parts of the body and to gently return to the chosen object of attention whenever the mind wanders off. Mindfulness-meditation practice can be basically understood as moment by moment awareness of the present experience [8]. This includes bodily, emotional, and mental aspects, such as sensations, feelings, and thoughts. The transformative effects associated with this method encompass increased bodily awareness, attention, the regulation of emotions, and changes in the perspective on the self [9,10]. These aspects may play an important role in the emergence and modulation of our sense of time. The capacity of trained mindfulness meditators to maintain the focus of attention on the bodily states and arising feelings, results, according to the insula model of consciousness awareness, in a slowing down of the subjective passage of time and a widening of the experienced moment in everyday experience [11].

With respect to the subjective experience of time in relation to meditation practice, one needs to differentiate between state- and trait-related findings. State-related findings refer to changes occurring directly during or immediately after a meditation session. In contrast, trait-related results indicate differences found when comparing experienced meditators with either non-meditating persons or with meditation novices regardless of any meditation practice. Experienced meditators report feelings of an extended present moment and a slowing down of the passage of time in everyday life, which may well result from states of mindfulness [12,13]. During meditation itself, however, experienced meditators report that they often get into a state of present awareness where subjective time and the sense of self are reduced after initially focusing on their bodies [14]. Only a few studies have attempted to empirically disentangle the relationship between meditation, mindfulness, and the present moment [15,16].

It is quite difficult to operationalize and measure the phenomenal experience of the present moment. Attempts have been made to demarcate a temporal-integration mechanism assumed to give rise to the feeling of *nowness* by generating important perceptual units of around two to three seconds’ duration [17,18]. It is debatable whether the experienced present moment stems from one integration mechanism or from several independent mechanisms with different durations. Nevertheless, they all are estimated to have an approximate range of a few seconds [19]. A paradigm providing support for temporal-integration processes comes from studies on the temporal dynamics of bi-stable figures. While observing a bi-stable figure, such as the Necker cube, two perceptual interpretations can be elicited, although the sensory stimulation remains unchanged. The stabilization in one perspective has been interpreted as being representative of perceptual units of present experience [17,20,21].

One recent study comparing 38 participants with and without meditation experience directly measured the effects of mindfulness proficiency on the duration of the present using the Necker cube. Results showed longer perceptual stability in meditators compared to controls in a task where they were asked to hold one perspective as long as possible. This result suggests an expanded present moment [15] and complements findings of an earlier study in which Tibetan Buddhist monks with extensive meditation practice were able to voluntarily prolong mental percepts of a different kind of visual bi-stable image [22]. Further evidence from psychological studies corroborates the notion that long-term effects of mindfulness meditation alter the sense of time. In a trait study, experienced meditators expressed less time pressure, more dilatation of past intervals, and a slower passage of time in general than non-meditators [13]. In another study looking at neurophysiological mechanisms underlying mindfulness, a longer production of time durations was accompanied by decreased frontal gamma activity in experienced meditators [23].

In the above-mentioned studies, the observed group differences can only be attributed to trait-like effects of mindfulness which, however, may not necessarily illuminate the mechanisms through which these differences emerge [24]. Very few systematic studies investigating the transient effects of meditation have provided evidence of state effects, i.e., changes in time perception in the seconds-to-minutes range right after meditation. One of the first systematic studies of a meditation-intervention effect on time perception in participants with no prior meditation experience was provided by Kramer and colleagues (2013) [25]. The authors compared participants’ performance on a temporal bisection task with stimuli ranging between 400 and 1600 milliseconds before and after meditation compared with a control intervention (listening to a recorded story). It was found that participants in the meditation condition overestimated durations post-meditation relative to pre-meditation, which suggests a subjective time dilation for short intervals after meditation. This finding corresponds with a more recent study applying a similar design with durations between four and eight seconds. A session of mindfulness meditation by experienced meditators produced a lengthening of interval durations compared to inexperienced meditators participating in the same session [26].

We know of no study which has investigated the length of the present moment during or right after mindfulness meditative states. In our study we aimed to fill this gap by assessing the effects of mindfulness meditation on the perception of the present moment operationalized by an auditory temporal-integration task. Temporal integration refers to the collection and processing of sensory stimulation over a certain period of time resulting in a coherent and unified percept [19]. A striking illustration of temporal integration within a time scale of a few seconds is the phenomenon of subjective rhythmization (SR). It refers to the phenomenon that, when listening to a sequence of identical tones at an isochronous pace, some of the tones are perceived as accented [27]. For instance, while listening to steady metronomic beats, perceived groups of beats automatically appear. Since the inter-beat intervals are isochronous and have equal intensity, the tones must be mentally linked, indicating that a temporal-integration process is taking place [28]. The extent of the temporal-integration phenomenon is limited to the respective time intervals. When the tempo of beats becomes too fast, i.e., when intervals are shorter than 200 milliseconds, the series of sounds cannot be perceived as separate, and no integration takes place [29]. When the frequency of presentation is too slow, the sequences are no longer merged into a perceptual segment, and a series of individual, unaccented beats is perceived. Although the magnitude of SR demonstrates some inter-individual variability, the upper limit is thought to lie at an inter-stimulus interval (ISI) of 1500–1800 milliseconds [30,31]. Szelag and colleagues (1996) [32] presented nine different metronome frequencies (from one up to five beats per second) and asked participants to integrate beats into larger units comprising two, three, or more beats by mentally accentuating them (i.e., every second, third, fourth, etc. beat). The participants reported how many beats they integrated into a unit at each metronome frequency. The integration-interval lengths, referring to the number of integrated beats multiplied by the ISIs between them, peaked at around 2.8 s for the lowest beat frequency and around 1.03 s for the highest. These findings and results using a similar paradigm in patients with brain injuries demonstrated that especially individuals with pre-frontal brain lesions show impairments in temporal integration [33,34]. This corresponds with the assumed temporal windows underlying the concept of the present moment upon which our study is based [35,36].

Our study presents first evidence of meditation-induced alterations of the present moment assessed with a temporal-integration task. We hypothesized that meditation would increase the width of the temporal-integration intervals. This study also investigated the role of mind-body interactions on the present moment by assessing the mediating effects of vagal modulation and breathing activity. The bidirectional connections between the brain and the autonomic nervous system are a favorable condition to assess the potential effects of meditation on interoceptive processes [37]. A number of neuroimaging studies have provided evidence for meditation-related changes in neural activity in brain areas associated with attention networks and the insular cortex [10,38]. It has already been shown that, by bringing attention to the present-moment experience, proficient meditators exhibited an increased activity of the right lateral pre-frontal cortex and the viscerosomatic regions, including the insula [39] (for a review, see Reference [40]). Another functional magnetic resonance imaging (fMRI) study exploring the perception of time with durations of three, nine, and 18 s demonstrated an accumulation of activity in the posterior insula [41]. The results supported the idea that the observed accumulator-type activity in the insular cortex could play a timekeeping role in the brain. Moreover, processing a time delay between external acoustic cues relative to the heart beats is related to activation in interoceptive regions, including the insula [6]. The encoding of temporal intervals lasting eight, 14, and 20 s has been associated with a progressive increase in cardiac periods [42]. Indices of heart-rate variability (HRV), indicating cardiac vagal control, were related to higher temporal accuracy with a stimulus duration around one second [43].

Given the close relationship between time perception, interoceptive awareness, and autonomic function, our goal is to further explore how this connection modulates our experience of the present moment. By comparing two groups of matched pairs of mindfulness meditators who either meditated or listened to a recorded story, we investigated: (a) Whether mindfulness meditation states are related to the perception of the present moment (as operationalized with the length of integration intervals of metronome beats after vs. before meditation) and (b) whether vagal tone (assessed with different indices of HRV) and the length of respiratory intervals mediate this effect.

## 2. Materials and Methods

### 2.1. Design

We performed a pre-post longitudinal study with two assessment-time points where the performance of meditators in a metronome task was compared before and after two different types of intervention (inter-subject comparison). We compared two different groups of paired meditators who were matched according to age (+/− 6 years), gender, education level (±1 of 5 levels), and meditation experience (level 1: 7–100 h; level 2: 100–200 h; level 3: over 200 h). A given individual either matched with an already-included participant (assigned to one of the two groups) or was randomly (by coin toss) assigned to one of two intervention conditions, namely a 10-min meditation (meditation) and a 10-min session of listening to a recorded story (story).

### 2.2. Participants

The study included 94 participants (54 females, 40 males; mean age: 25 years (SD = 3.7 years); age range: 18–37 years; 90 right-handed; after outlier analysis: *n* = 91) with mostly low-to-moderate experience in mindfulness meditation. As a minimum requirement, an individual had to have completed a guided meditation course (i.e., a formal meditation course instructed and guided by a qualified coach) at least once and have practiced at least seven h within the last year (range of h of life-time experience: 7–3262 h; median: 94 h; mean: 210 h; SD: 468 h). This minimum requirement was to ensure that participants had some basic understanding of mindfulness meditation practice and would be able to perform it with ease. Performance on a temporal-processing task has been shown to be influenced by meditation in student participants without prior meditation experience [25]. Our subjects were recruited by advertisements in meditation centers, on online platforms for student jobs at the University of Freiburg, and by word of mouth. Participants were only included if they were familiar with a meditation form which stressed awareness of the present moment (e.g., mindfulness meditation, Vipassana meditation, or Soto Zen). The two pair-matched groups, each consisting of 47 subjects, were created and subjected to the experimental condition (a guided meditation session lasting 10 min) and the control condition (listening to a story for 10 min). The present metronome task was one of three different experimental tasks conducted similarly on three consecutive days in a randomized order. The results of the other two tasks will be reported elsewhere. Participants were fluent in German and, as self-reported in a check list, were healthy and denied any history of neurological or psychiatric disorders. They received a financial compensation (€50 for all sessions combined) for taking part in the three study sessions conducted on three consecutive days and lasting about one hour each. All participants gave written informed consent prior to data collection. The study was approved by the Ethics Committee of the German Society of Psychology (Deutsche Gesellschaft für Psychologie) and the approval code is: MW 032015_Zeit.

### 2.3. Apparatus and Physiological Recordings

The computerized task was run on a Pentium 4 Dell optipLEx 755 PC with a Samsung SyncMaster 757 DFX monitor (17 inch, 85 Hz refresh rate). The metronome beats were created using the Metronom-Plus software (M and M Systeme, 2009). The audio recordings (meditations and stories) were played back on the open-source media player VLC (VideoLAN, 2001). The acoustic stimuli were presented using the Beyerdynamic DT 990 PRO open dynamic headphones at a comfortable hearing level.

The electrocardiogram (ECG) was recorded with the eMotion FAROS 360° mobile device (Mega Electronics Ltd., Kuopio, Finland), a three-channel ECG recorder. This device also records breathing activity with an integrated 3-D accelerometer placed on the chest. The breathing signal obtained with an accelerometer has proven to be similar to the signal measured with an inductive band (Hung et al., 2008). ECG data was acquired at a 500-Hz sampling rate using two disposable Ag-AgCl electrodes which were positioned according to a modified Lead II Einthoven configuration, one placed centrally under the right clavicle and the other on the 11th left intercostal space. The electrodes were connected to the eMotion FAROS 360° mobile device. Breathing data was acquired at a 400–Hz sampling rate and at the dynamic range +/−4 g (1 g = 9.81 m/s²). The accelerometer was positioned at the level of the 3rd intercostal space on the right side of the sternum. Participants sat with their hands placed on their thighs and leaned back against the backrest of a comfortable chair.

### 2.4. Instruments

#### 2.4.1. Freiburg Mindfulness Inventory-14 (FMI-14)

The FMI-14 [44] assesses self-reported mindfulness with two factors: ‘Presence’ and ‘acceptance’. The former factor considers the ability to be in the present moment (“I feel connected to my experience in the here-and-now”), and the latter concerns a non-judgmental attitude (“I am able to smile when I notice how I sometimes make life difficult”). The answers are scored using a four-point scale ranging from ‘rarely’ to ‘almost always’. The FMI has been psychometrically validated on the basis of classic test theory and Rasch analysis [45,46].

#### 2.4.2. Metronome Task

The task consisted of unaccented isochronous metronome beats (auditory clicks). Each single beat lasted 19 milliseconds. Sequences of beats contained the following frequencies (beats per second): 0.333, 0.5, 0.75, 1, 2, and 3 Hz, representing 20, 30, 45, 60, 120, and 180 beats/min, respectively. Participants were asked to listen to the sequences and to try to let an accentuated rhythmic pattern subjectively emerge spontaneously (e.g., 1-2, 1-2, or 1-2-3, 1-2-3, etc.). As soon as they perceived the accentuated rhythmic pattern, they were asked to tell the experimenter how many beats the rhythmic pattern contained before the next stimulus was played. The duration of the accentuated rhythmic pattern can be calculated (temporal integration interval as a measure of the experienced moment) from the number of automatically integrated beats into one rhythm group. The dependent variable ‘temporal integration interval’ (TI) corresponds to the width of the integrated beats from onset to offset into one temporal unit (Gestalt). For example, for a participant who reported hearing a grouping of two beats at an ISI of 3 s, the TI would then be 3 s; for another participant reporting hearing 3 beats at the same frequency, the TI would be 6 s.

The experiment itself consisted of a training phase and a test phase. During t1 participants accomplished the training phase and a test phase during t2 only the test phase was presented. The training phase consisted of three stimulus frequencies (0.5, 1, and 2 Hz, corresponding to 30, 60, 120 bpm) presented twice in random order, resulting in six trials. The test phase was composed of six stimulus frequencies (0.333, 0.5, 0.75 1, 2, and 3 Hz, corresponding to 20, 30, 45, 60, 120, and 180 bpm), each of which was presented four times in random order, resulting in 24 trials. The median of responses per inter-stimulus interval (ISI) was taken as the measure of a central tendency for a given frequency for each of the four trials. Across all ISIs, the sum of the TIs can be calculated as the area under the curve (AUC) to represent the overall size of integration across intervals.

#### 2.4.3. Interventions

The meditation and control interventions were delivered by audio recordings in German lasting 10 min each (three guided meditations and three stories). They were extracted from professionally-guided meditation recordings and audiobooks (see Supplementary Materials for the transcripts in German and the English translations). The meditations were mindfulness-based meditations designated to focus attention either on breathing [47], on the whole body [47] or on both [48]. The stories were taken from three different audiobooks: “Der Umweg” (The Detour) [49], “Mein Venedig” (My Venice) [50] and “Das Meer am Morgen” (The Sea in the Morning) [51]. We decided to choose stories which involved neutral third-person narratives to keep the attentional focus as far as possible from bodily-related sensations. In contrast, the focus of attention in the meditation recordings was internally (body) oriented. We thus ensured that both kinds of interventions demanded an analogous amount of attention, but focused attention on different locations (body-self in meditation vs. body-distant in audiobooks).

### 2.5. Procedure

The investigation took place in a laboratory room at the Institute for Frontier Areas of Psychology and Mental Health in Freiburg, Germany. The experiment was carried out with artificial light (fluorescent lamp); lighting conditions were kept constant. Participants sat in a comfortable armchair. The experimenter remained in the room during the whole testing period, including the intervention session.

Participants gave written informed consent and filled in questionnaires about socio-demographic variables, meditation experience, and the FMI questionnaire at the beginning of the first session. A series of questions concerning the intake of coffee, tea, alcohol, nicotine, and food during the hours before the experiment were asked at the beginning of the session. Quality (with a scale ranging from excellent to extremely bad) and quantity (hours) of sleep the previous night were also assessed. Electrodes were attached, and the electrocardiogram (ECG) and breathing activity were recorded during a 10-min baseline measure during which participants were requested not to move during the recording phase and to breathe normally. One of the three psychophysical tasks was then presented (t1) followed by the 10-min intervention (meditation, story), during which the heart and breathing rates were recorded. The metronome task was repeated (t2) after the intervention.

### 2.6. Data Reduction, Statistical Approach

ECG signals and time intervals between successive R-peaks (RR intervals) were automatically extracted using Kubios HRV Analysis Software 2.0 (Kuopio, Finland). They were visually examined to control for ectopic and missing beats and automatically corrected afterwards choosing the appropriate threshold level for artefact correction available in Kubios. The signal was subsequently detrended using the smoothness-of-priors method [52]. Because our focus of interest was on vagal modulation, we computed the root mean square of successive differences (RMSSD) and the high-frequency (HF) spectrum according to the recommendations for heart-rate variability (HRV) assessment by Laborde et al. (2017) [53]. Although time-domain (i.e., RMSSD) and frequency-domain (i.e., HF component) measurements of HRV have been shown to correlate [54,55], there is some evidence that these indices may not strictly have the same biological meaning [56].Therefore, we proceeded to study vagal-mediated changes on HRV using both indices. RMSSD is a time-domain HRV reflecting short-term variability of the heart rate (HR). It represents the most common time-domain measure used to estimate parasympathetic activity [57,58] and is reported to be independent of respiratory effects [59,60]. Concerning HRV parameters in the frequency-domain, we computed the Fast Fourier Transformation (FFT) to calculate the relative power of the HF spectrum (HF[%]: HF[m²]/total power[m²] × 100%) of the RR intervals series. The HF band between 0.15 and 0.40 Hz is also thought to reflect vagal activity [61] and, contrary to RMSSD, is believed to be affected by breathing when it is below 9 or above 24 cycles per minute [61,62]. To ensure that the HF components represented a reliable estimation of the vagal activity, we used the obtained breathing-intervals average (BR) to control for respiratory rates below 9 or above 24 cycles per minute, which reflects the 0.15 and 0.40 Hz band regarding HFs. Participants whose breathing rate exceeded these limits were excluded from the frequency domain HRV analysis.

The peak-detection function available in AcqKnowledge 3.7.2 was used to extract the respiration periods from the breathing signal. Breathing signals were visually examined, and artefacts were manually corrected. Respiratory periods were resampled at 15.625 samples per second employing a linear interpolation method. A band-pass filter using a Hanning window and a low- and high-frequency cut-off of 0.08 Hz and 0.42 Hz respectively was applied. Breathing intervals were computed and averaged over each 10-min recording session.

**Figure 1:**
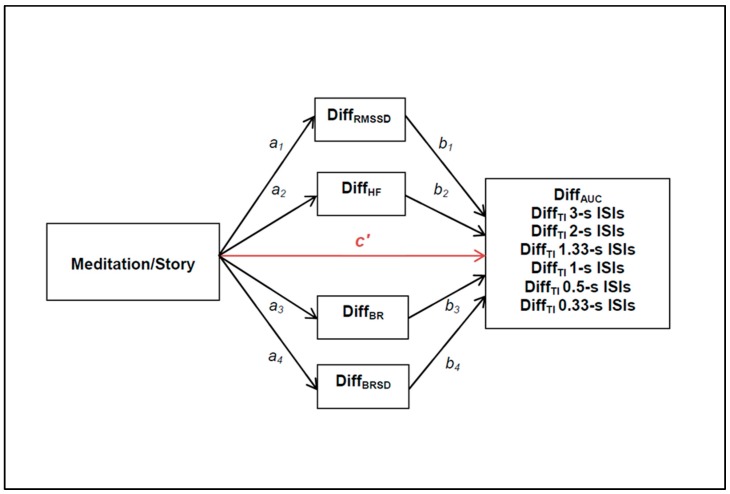
Illustration of the multiple-mediation model applied here. The possible associations between the type of intervention (X: meditation vs. story) and the psychophysical variables (Y: Diff_AUC_, Diff_TI_ 3 s ISI, Diff_TI_ 2 s ISI, Diff_TI_ 1.33 s ISI, Diff_TI_ 1 s ISI, Diff_TI_ 0.5 s ISI, Diff_TI_ 0.33 s ISI), either directly (***c*’ path in red color**) or through the physiological mediating variables (*a_i_b_i_* paths; Diff_RMSSD_, Diff_HF_, Diff_BR_, Diff_BRSD_), are pictured with arrows.

Because one of the main aims of this study was to understand how a meditation intervention influences temporal processing, i.e., to discern the mechanisms through which these influences occur, we used a path-analytic model (PROCESS) [63] to test the significance of potential mediation effects in our group of variables. Mediation analysis reveals how a predictor variable X affects a dependent variable Y, either directly or via one or more mediating variables M. Such a method should only be used when the order of variables can be longitudinally established, which was the case since the present study had two time points. We hypothesized that the meditation induction could affect temporal processing as captured by the psychophysical tasks, either directly or indirectly through physiological factors (ECG and breathing parameters). Figure 1 depicts the multiple mediation model employed here. The c’ path represents the direct effect of *X*→*Y*, whereas, *a_i_* and *b_i_* depict the path coefficients for *X*→*M* and *M*→*Y* effects, respectively. The *a_i_b_i_* path represents the indirect effect of *X*→*Y* through mediators *M,* which are calculated as the product of *a_i_* and *b_i_*. The *c* path can be expressed as the difference between the total effect of *X*→*Y* (*c* = *c*’ + *a_i_b_i_*) and the indirect effect of *X*→*Y* through *M*, namely, *c*’ = *c* − *a_i_b_i_*. In our design, *X* represents the two different interventions (independent variables: meditation vs. story), while *Y* and *M* variables are calculated as the difference (Diff) between t2 and t1 for the psychophysical task (*Y* dependent variables: Diff area under the curve (Diff_AUC_) as combined variable across all ISIs, Diff temporal integration (Diff_TI_) for each individual ISI) and the difference between the recordings during the intervention session and the baseline measure for the physiological variables (*M* mediator variables: Diff for RMSSD, HF, BR mean (BR), BR standard deviation (BRSD), respectively.

The PROCESS software enables testing for indirect effects using either a bootstrap approach or a normal-theory approach (e.g., the Sobel test). The multiple-mediation model used here includes N = 5000 bias-corrected (BC) bootstrap resamples and was set at a 95% confidence level. When zero is not in the 95% confidence interval, the indirect effect is considered to be significantly different from zero at *p* < 0.05 (two tailed) [64]. By applying this procedure, as recommended by MacKinnon, Lockwood, and Williams (2004) [65], we aimed at avoiding power problems due to non-normal or asymmetric sample distributions. The SPSS macros are available at Andrew F. Hayes’ website: http://afhayes.com/spss-sas-and-mplus-macros-and-code.html.

According to Baron and Kenny (1986) [66], the first assumption for conducting mediation analyses is the existence of a causal relation between X and Y, i.e., the total effect must be significant (*c* ≠ 0). However, opposing arguments allowing the use of mediation procedures without requiring significant total effects have been proposed [67]. For mediation effects to be present, a total effect X→Y is assumed to be initially present [68]. No such assumption of a total effect of X→Y is necessary for the assessment of indirect effects [64,69]. Accordingly, it is possible to find a significant indirect effect even when there is no significant total effect. The variable for individual ISI ‘Diff_TI_’ was non-normally distributed. To attain or approach normal distributions, this variable was log (ln) transformed before accomplishing the statistical analysis.

### 2.7. Outlier Analysis

Eight subjects were completely excluded from the data analyses because they fell asleep during one of the interventions (5 in the meditation group, 3 in the story group). We also excluded participants who did not hear any groups of beats when listening to the isochronous metronome sequences (one participant in the meditation group) and who were assumed to count the beats, thereby misinterpreting the instructions (one participant in the story group integrated more than 8 beats).

The rationale of using 8 beats as an upper limit of grouping comes from studies on subjective rhythmization [70] and on spontaneous perception in synchronization tasks [71], showing that groups of 2, 4, and a maximum of 8 beats are preferred. Partly based on the observations of Båath (2015) [31] and Fraisse (1982) [30], but also taking into account conceptualizations on the present moment stemming from perceptual units of around two to three seconds’ duration [17,18], we also computed analyses additionally excluding integration intervals longer than 3-seconds duration (in the following ‘max. 8/3 s’ criterion). The grouped beats may integrate a maximum interval of TI = 3 s from onset to offset of the integrated beats. For example, for the ISI = 3 s, a maximum grouping of two beats is allowed, since a grouping of 3 beats (TI = 6 s) is the first to already exceed the 3-second limit. For the ISI = 2 s, also only a maximum grouping of two beats is allowed, with a grouping of 3 beats already integrating TI = 4 s. Further, maximum groupings are 3 for ISI = 1.333 s, 4 for ISI = 1 s, 7 for ISI = 0.5, and 8 for ISI = 0.333 s, respectively (for an overview, see Appendix A, Table A1 and Table A2).

## 3. Results

### 3.1. Sample Description

Descriptive statistics are summarized in Table 1. As expected after a matching procedure, there were no differences between groups with respect to age, gender, educational level, and meditation experience. The levels of self-reported mindfulness as measured with the FMI sub-scales ‘acceptance’ and ‘presence’ did not differ significantly between groups.

### 3.2. Descriptive Analysis

The groupings most commonly reported over all trials (the two conditions, pre- and post- intervention) were two and four, three and eight were less commonly reported (Table A3 in Appendix A shows the percentage of responses for each ISI). This corresponds to typical groupings found in the literature [31].

### 3.3. Mediation Analysis

A series of mediation analyses was conducted. The path analyses were computed between groups (X = meditation vs. story), for each dependent variable separately (Diff_AUC_ and Diff_TI_ at: 0.33, 0.50, 0.75, 1, 2 and 3-second ISI), and using four potential intervening variables (Diff_RMSSD_, Diff_HF_, Diff_BR_ and Diff_BRSD_) (see Figure 1). The analyses were conducted on the total sample (*n* = 84). The following reported coefficients are non-standardized.

Results from mediation analyses with the whole sample (*n* = 84) showed a positive relation between meditation and Diff_RMSSD_ (*p* < 0.05), Diff_BR_ (*p* < 0.001) and Diff_BRSD_ (*p* < 0.001) (*a* path) indicating that participants who meditated, as compared to those who listened to the story, had a higher HRV in the time domain, as well as longer breathing intervals and a higher breathing variability. Means and standard deviations (SD) corresponding to the *a* path coefficients are shown in Table 2, where the mean and SD of the physiological variables separated for the two intervention groups (meditation, story) are listed for the baseline condition, the intervention session, and the difference (Diff) between intervention and baseline (the Diff variables are used in the mediation analyses).

**Figure 2:**
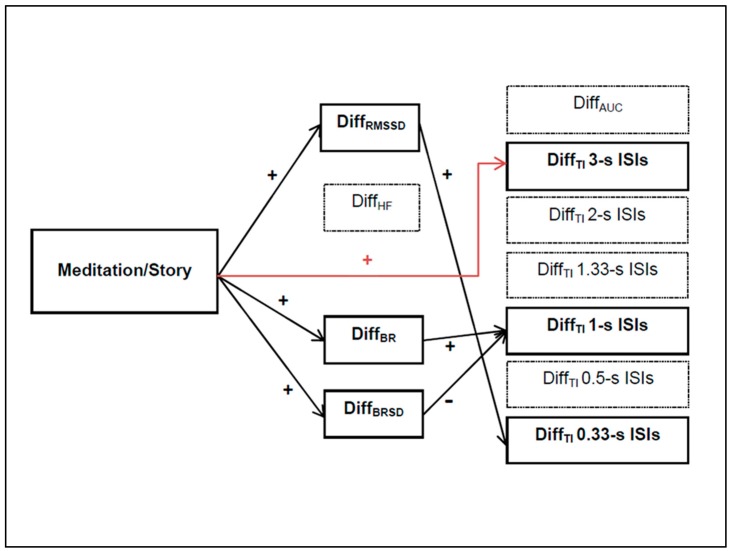
Illustration of the significant *X*→*M* relation (*a* path) and the significant direct effect (***c*’ path in red color**) and indirect effects (*ab* paths) obtained by the mediation analysis with *n* = 84. The direction of the effects is pictured with **+** for a positive relation and **−** for a negative one.

**Figure A1:**
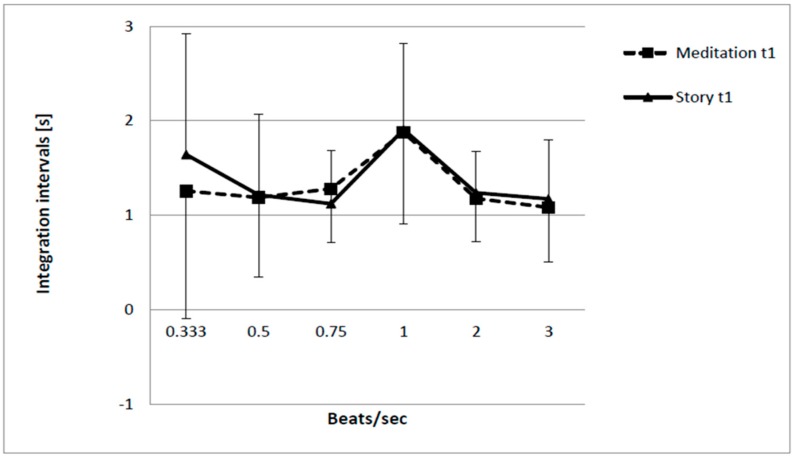
Perceived beats (integration interval) as a function of the metronome frequency (beats/sec) before meditation (t1).

**Figure A2:**
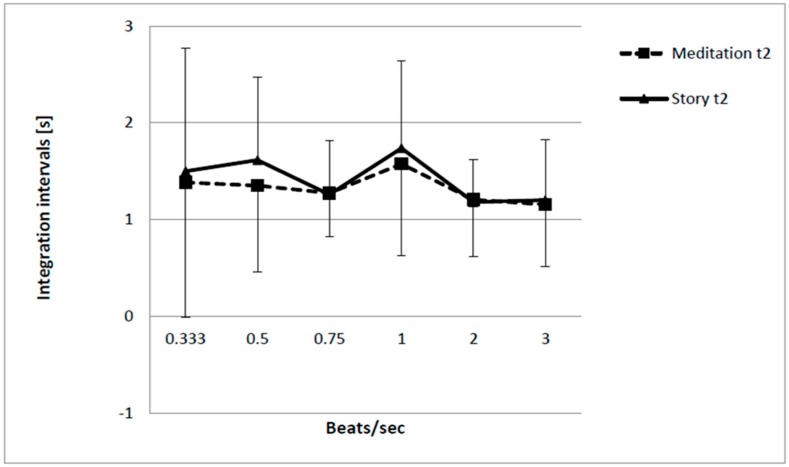
Perceived beats (integration interval) as a function of metronome frequency (beats/sec) after meditation (t2).

There was a direct effect (*c*’ path) of the type of intervention on the Diff_TI_ at 3 s (*b* = 0.21, *t*(64) = 2.30, *p* < 0.05). When controlling for the physiological variables, participants who meditated showed relative expanded TIs at 3-second ISIs as compared to those who listened to the audio story. Moreover, the specific indirect effects (*ab* path) of the type of intervention through Diff_BR_ (*b* = 0.13, CI: 0.04; 0.26; *p* < 0.05) and Diff_BRSD_ (*b* = −0.14, CI: −0.28; −0.06; *p* < 0.05) on Diff_TI_ at 1-second ISI, as well as through Diff_RMSSD_ (*b* = 0.02, CI: 0.002; 0.04; *p* < 0.05) on Diff_TI_ at 0.33-second ISIs, were significant. These results suggest that longer breathing intervals during meditation lead to longer temporal-integration intervals at 1-second ISIs, and greater breathing variance produces a shortening of temporal integration at the same frequency. Additionally, the higher the HRV in the time domain, the longer the integration intervals at 0.33 s ISIs. An overview of the significant results can be seen in Figure 2; no other paths of the model produced significant results (see Table 3). For a graphical depiction of the integration intervals over the different metronome frequencies separately shown for the two conditions pre- (t1) and post (t2) intervention, see Appendix A, Figure A1 and Figure A2.

### 3.4. Relationship Between Trait-mindfulness and the Metronome Task

No significant correlation between self-reported mindfulness, as measured with the subscales ‘presence’ and ‘acceptance’ from the FMI questionnaire, and the AUC at t1, as well as with the independently calculated Diff_AUC_ for both groups were found (*p* > 0.05). The relationship between self-reported mindfulness and the individual TIs at the different metronome frequencies was also non-significant (for all frequencies *p* > 0.05).

## 4. Discussion

The present study investigated how states of mindfulness meditation and autonomic activity modulate the experience of the present moment. Two groups of mindfulness meditators, who either meditated or listened to a recorded story, performed an auditory temporal-integration task before and after the intervention. We recorded indices of vagal and respiratory activity during the intervention sessions to determine whether meditation-induced changes at that level influence the time characteristic of the present moment.

We revealed clear effects of meditation on physiological parameters. Participants who meditated showed greater heart-rate variability in the time domain, as well as longer breathing intervals and higher breathing variability as compared to those participants who listened to the recorded stories. Regarding the psychophysical task, meditation induction led to longer integration intervals at the slowest metronome frequency (3-second ISIs). With respect to autonomic activity indices, higher vagal activity in participants under meditation induction was related to widened integration intervals at 0.33-second ISIs, the fastest frequency. Participants who meditated and had longer respiratory periods also displayed longer integration intervals at 1-second ISIs. Higher breathing-rate variability, on the contrary, was associated with a shortening of the integration intervals for the same frequency.

First studies investigating the effects of meditative states on time perception in the seconds range have shown a subjective dilation of interval durations after meditation [25,26]. In our study, participants who meditated integrated more metronome beats into a perceptual unit as compared to those meditators who listened to recorded stories, but only as a direct effect at the assumed upper limit of the present (3-second ISIs). A tentative interpretation is that the upper limit of the present moment was expanded after meditation. Our study is probably the first to investigate the effects of meditative states on temporal integration in the seconds range by using the metronome paradigm and controlling for physiological effects. According to phenomenological arguments, the events of experience occurring *now* are surrounded by memories of what just happened and anticipation of subsequent actions [72]. The experience of a moment is extended and contains memory (past component) and expectation of what is about to happen (future component).The heightened awareness of the present moment attained by highly experienced meditators leading to the loss of bodily sensations (selflessness) has been described as diminishing the past and future components surrounding the present and provoking the feeling of timelessness [73]. This is a state that only very experienced meditators can attain. In our study, states of mindfulness led to a lengthening of the temporal integration intervals for the slowest metronome frequency. The participants in the present study on average had only low-to-moderate meditation experience and meditated for only 10 min. The state of meditation our participants achieved was one with an increased bodily awareness as mindfulness experience.

Taken together, the observed associations between HRV and breathing activity and the length of the temporal-integration intervals in the current study complement prior observations of a relationship between vagally-mediated changes in cardiac activity and time perception [42,43,74]. These results also corroborate Craig’s theory that integration and modulation of bodily signals (i.e., cardiorespiratory activity) may affect temporal processing [1].

An early study investigating temporal-integration mechanisms in patients with acquired brain injuries revealed that patients with prefrontal-lobe damage in the left hemisphere had difficulties integrating metronome beats [33]. They found that these patients relied on a counting strategy and not on automatic temporal integration like the control patients did. These findings fit well with Thayer and Lane’s (2000) neurovisceral-integration model [75]. A continuous brain-periphery communication is assumed that links the heart, cortical (i.e., prefrontal areas) and sub-cortical regions. Indices of HRV have been identified as reflecting the inhibitory effect of prefrontal-subcortical networks on sympathoexcitatory subcortical circuits [76]. More strikingly, higher resting-HRV levels have been associated with the efficient functioning of the inhibitory prefrontal-subcortical circuits [77] as revealed by better executive functioning in a sustained-attention test [78]. Higher HRV, through an improvement in executive functioning, may have enabled participants to maintain their focus of attention (i.e., sustained attention) longer, leading to the lengthening of the integration intervals at the fastest metronome frequency.

There are several limitations of our study which should be addressed in future studies. Our design comprised matching pairs of meditators according to the criteria age, gender, education level, and meditation experience. We could have used a randomization procedure to more strongly control for possible confounders between experimental groups and applied a stratification method for controlling the above variables. Moreover, our effects are modest. Only one direct effect of meditation states was found, and this effect was only significant for the longest metronome frequency assumed to represent the upper limit of the present moment. The indirect effects were rather unsystematic. One could argue that the three frequencies represented special intervals: The upper (3 s) and lower (0.33 s) boundaries at which integration is possible at all, and was therefore especially sensitive to effects and the socially-learned interval of clock time (1 s). We consider our approach to be a first step in assessing temporal-processing mechanisms in an experimental operationalization of present-moment experience. Only meditators with extensive experience should be included in future studies. In our study, only 44 % of the participants had 100 or more hours of meditation experience. More participants could be tested if the focus were placed on more experienced individuals. We used prerecorded meditation sessions in our study to achieve a standardized protocol. These are helpful for individuals who are less experienced. Experienced meditators in future studies should be allowed to use their own meditation techniques; meditation should not be induced by a recording. This would allow meditators to perform a longer meditation session. Another issue of exploring mindfulness-meditation-related techniques remains the difficulty in differentiating the features of the experience targeted (i.e., through instructions) from the actual phenomenological state being attained [24]. Controlling for the targeted state and for the degree to which this state has been reached could shed some light on how meditation influences time perception.

A growing number of studies of altered states of consciousness in general and during meditation in particular have been conducted in the fields of psychology and neuroscience [79,80]. The scientific study of meditative states, describable as a change in present experience, is in its infancy. These attempts should reveal what constitutes everyday consciousness and what happens during altered states of awareness, essentially probing the mind-body problem. Our study constituted a first attempt to measure meditative states in meditation-experienced individuals and disclosed one direct effect of meditation and several indirect effects through changes in physiology. Our study is in line with an increasingly popular field of research.

## Figures and Tables

**Table 1:** Descriptive statistics of the two study groups (*n*= 84).

Variable	Meditation Group (*n* = 41)	Story Group (*n* = 43)	*p*-Value ^a^
Age (mean ± SD)	25 ± 3.7	25 ± 3.4	0.937
Gender (female (%))	25 (29.8)	25 (29.8)	0.791 ^b^
Educational level			0.953 ^b^
Secondary school (n (%))	1 (1.2)	1 (1.2)	
High school (n (%))	27 (32.1)	27 (32.1)	
University degree (n (%))	13 (15.5)	15 (17.9)	
Meditation experience (mean ± SD)	223 ± 511	218 ± 484	0.963
Trait-mindfulness (FMI)			
Acceptance (mean ± SD)	24 ± 3.4	23 ± 2.6	0.204
Presence (mean ± SD)	19 ± 4.2	18 ± 2.1	0.203
Sum (mean ± SD)	42 ± 4.8	41 ± 3.9	0.213

^a^ t-Test if not otherwise indicated; ^b^ Chi- Square Test.

**Table 2:** Descriptive statistics of the two study groups within the whole sample (*n* = 84) for the resting state, the interventions (Meditation vs. story) and the *a* path calculated as the difference in variables between intervention and resting state (Diff.).

Variable	Meditation Group (*n* = 41)	Story Group (*n* = 43)	*p*-Value
Resting RMSSD (mean ± SD)	36.8 ± 22.6	35.4 ± 22.3	0.774
Resting HF (mean ± SD)	48.7 ± 19.8	48.4 ± 21	0.950
Resting BR (mean ± SD)	4.3 ± 0.86	4.2 ± 0.96	0.692
Resting BRSD (mean ± SD)	0.90 ± 0.47	0.98 ± 0.56	0.480
Intervention RMSSD (mean ± SD)	48.4 ± 33.7	38.9 ± 23.3	0.139
Intervention HF (mean ± SD)	41.1 ± 21.3	35.9 ± 17.9	0.263
Intervention BR (mean ± SD)	5.1 ± 1	4.1 ± 0.88	0.000 ***
Intervention BRSD (mean ± SD)	1.4 ± 0.65	1.1 ± 0.62	0.006 **
Diff. RMSSD (mean ± SD)	11.4 ± 18.3	3.4 ± 11.8	0.019 *
Diff. HF (mean ± SD)	−7.5 ± 26.3	−9.1 ± 24.7	0.798
Diff. BR (mean ± SD)	0.73 ± 0.81	−0.17 ± 0.69	0.000 ***
Diff. BRSD (mean ± SD)	0.50 ± 0.55	0.03 ± 0.46	0.000 ***

* Significant coefficients: * *p* < 0.05, ** *p* < 0.01, *** *p* < 0.001 marked **in bold**.

**Table 3:** Summary of mediation results for the metronome task with the whole sample (*n* = 84).

X Independent Variable	M Diff Mediating Variables	Y Diff Dependent Variables	Effect of X→M (a)	Effect of M→Y (b)	Specific Indirect Effects (a, b)	Direct Effect X→Y (c’)	Total Effect (c)	Type of Effect
	RMSSD	Area under the curve AUC	8.59 *	−0.00	−0.00	0.04	0.01	**none**
	HF		1.56	0.00	0.00			
	BR mean		0.80 ***	0.07	0.06			
	BR SD		0.49 ***	−0.19	−0.09			
**Meditation vs. Story**	RMSSD	Integration interval at 3 s ISI	8.87 *	−0.00	−0.05	0.21 *	0.06	**Direct effect**
	HF		3.02	0.00	0.00			
	BR mean		0.77 ***	−0.12	−0.09			
	BR SD		0.45 ***	−0.01	−0.00			
	RMSSD	Integration interval at 2 s ISI	8.31 *	0.00	0.01	−0.11	−0.06	**none**
	HF		0.57	0.00	0.00			
	BR mean		0.82 ***	0.03	0.02			
	BR SD		0.50 ***	0.01	0.01			
	RMSSD	Integration interval at 1.33 s ISI	9.67 *	0.00	0.01	−0.01	0.01	**none**
	HF		−1.86	−0.00	0.00			
	BR mean		0.75 ***	0.03	0.01			
	BR SD		0.53 ***	−0.00	−0.00			
	RMSSD	Integration interval at 1 s ISIs	8.68 *	−0.00	−0.00	0.00	−0.01	**Indirect effect**
	HF		−1.00	−0.00 *	0.00			
	BR mean		0.77 ***	0.17 ***	0.13 *			
	BR SD		0.49 ***	−0.29 **	−0.14 *			
	RMSSD	Integration interval at 0.5 s ISIs	9.68 *	0.00	−0.00	0.05	0.03	**none**
	HF		1.95	0.00	0.00			
	BR mean		0.77 ***	0.00	0.00			
	BR SD		0.48 ***	−0.06	−0.02			
	RMSSD	Integration interval at 0.33 s ISIs	8.73 *	0.00 *	0.01 *	0.00	−0.00	**Indirect effect**
	HF		1.92	0.00	0.00			
	BR mean		0.79 ***	0.00	0.00			
	BR SD		0.48 ***	−0.06	−0.03			

* Significant (non-standardized) coefficients: * *p* < 0.05, ** *p* < 0.01, *** *p* < 0.001 marked **in bold**.

## Supplementary Materials

The following are available online at https://www.mdpi.com/2076-328X/9/5/51/s1, Excel E1: data_metronome; Word W1: Interventions in German and English.

## Appendix A

**Table A1:** Temporal integration intervals (TI) depending on ISI and integrated beats Individual responses were not used in the analysis when they exceeded an upper temporal integration limit of 3 s.

Integrated Beats	1	2	3	4	5	6	7	8
**ISI = 3 s**	0 s	3 s	6 s (excluded)	excluded	excluded	excluded	excluded	excluded
**ISI = 2 s**	0 s	2 s	4 s (excluded)	excluded	excluded	excluded	excluded	excluded
**ISI = 1.333 s**	0 s	1.33 s	2.66 s	3.99 s (excluded)	excluded	excluded	excluded	excluded
**ISI = 1 s**	0 s	1 s	2 s	3 s	4 s (excluded)	excluded	excluded	excluded
**ISI = 0.5 s**	0 s	0.5 s	1 s	1.5 s	2 s	2.5 s	3 s	3.5 s (excluded)
**ISI = 0.333 s**	0 s	0.33 s	0.66 s	0.99 s	1.33 s	1.665 s	1.99 s	2.33 s

**Table A2:** Excluded trials per grouping.

ISI	> 3 s Criterion	> 8 Criterion	SUM (Both Criteria)	% of Trials
0.333	0	4	4	0.53
0.5	93	2	95	12.63
1	29	1	30	3.98
1.333	201	0	201	26.72
2	164	0	164	21.80
3	93	0	93	12.36
	580	7	587	13.01
% of trials	12.86	0.15	13.01	

**Table A3:** Summary of reported groupings in the metronome task; grouping of 1 corresponds to no integration of beats.

Grouping	% of Trials	Peak ISI
1	18.54	3
2	35.44	1.333
3	8.58	0.5
4	27.70	0.5
5	0.95	0.333
6	1.62	0.333
7	0.53	0.333
8	6.48	0.333
>8	0.16	0.33
