# Supplementary material for: Meditation-Induced States, Vagal Tone, and Breathing Activity Are Related to Changes in Auditory Temporal Integration
Source: Behav Sci (Basel). 2019 May 7;9(5):51. doi: 10.3390/bs9050051 (PMC6562910; doi:10.3390/bs9050051)
Supplement: Supplementary file 1 [file behavsci-09-00051-s001.zip › Interventions in German and English.docx]

Interventions in German language

Stories

Das Meer am Morgen by M. Mazzantini, 2011

Farid hat das Meer nie gesehen, ist nie hineingetaucht. Er hat es sich oft vorgestellt: sternenübersäät, wie der Mantel eines Paschas, blau, wie die blaue Mauer der toten Stadt. Er hat die versteinerten Muscheln gesucht, die vor Jahrmillionen verschüttet wurden, als das Meer in die Wüste kam, hat den Eidechsenfischen nachgespürt, die unterm Sand schwimmen, er hat den Salzsee gesehen und den Bittersee und die silbrigen Dromedare, die sich wie ramponierte Piratenschiffe fortbewegen.

Er wohnt in einer der letzten Saharaoasen. Seine Vorfahren gehörten zu einem Stamm nomadisierenden Beduinen. Sie schlugen ihre Zelte in Kadis auf, in Pflanzenbewachsenen Flussbetten. Die Ziegen weideten. Die Ehefrauen kochten Essen auf glühend heißen Steinen. Sie hatten die Wüste nie verlassen. Es herrschte ein gewisses Misstrauen gegenüber den Leuten von der Küste. Händler, Seeräuber.

Die Wüste war ihr zu hause. Weit und grenzenlos. Ihr Sandmeer. Von Dünen gefleckt, wie das Fell eines Jaguars. Sie besaßen nichts. Nichts als Fußspuren, auf die sich der Sand legte. Die Sonne bewegte die Schatten. Sie waren es gewohnt, dem Durst zu trotzen und wie Datteln auszudorren, ohne zu sterben. Ein Dromedar bahnte ihnen den Weg, ein langer schiefer Schatten. Sie verschwanden in den Dünen. Für die Welt sind wir unsichtbar, doch nicht für Gott. Mit diesem Gedanken im Herzen zogen sie umher.

Im Winter ließ der Nordwind, der über den felsigen Ozean kam, den wollenen Bergan am Körper steif werden und die Haut blutleer, wie die der Ziegen auf den Trommeln, klammerte sich an die Knochen. Uralte Zaubereien fielen vom Himmel. Die Sandverwerfungen waren messerscharf. Die Wüste berühren hieß sich verletzen. Die Alten wurden begraben, wo sie starben, der Stille des Sandes überlassen. Die Beduinen zogen weiter, Stofffetzen in Weiß und Indigo.

Im Frühling entstanden neue Dünen, rosig und blass. Jungfrauen aus Sand. Der glühende Gibli kam mit dem kratzigen Ächzen des Schakals. Hier und dort zwickten kleine Windkringel, wie umherfahrende Geister den Sand, dann Böen im Tiefflug, scharf wie Krummsäbel. Eine aufgeweckte Armee. Die Wüste erhob sich in Sekundenschnelle und verschlang den Himmel. Eine Grenze zum Jenseits gab es nicht mehr. Die Beduinen beugten sich unter Last des grauen Sturms, suchten Schutz hinter dem Körper kauernder Tiere, wie unter dem Mantel eines alten Fluchs.

Dann waren sie geblieben. Sie hatten aus Lehm eine Mauer gebaut und Weideland umzäunt. Im Sand waren Radspuren. Manchmal kam in dieser Gegend eine Karawane vorbei. Sie lagen an der Route der Händler aus Schwarzafrika, die ihren Weg zum Meer durch die Wüste abkürzten. Sie hatten Elfenbein, Harze, Edelsteine und gefesselte Menschen dabei, die in den Häfen der Cyrenaica und Tripolitaniens als Sklaven verkauft werden sollten. Die Händler stärkten sich in der Oase. Sie aßen, tranken. Eine Stadt war entstanden. Mauern aus getrocknetem Lehm, geflochtenen Seilen ähnlich, Dächer aus Palmwedeln. Die Frauen wohnten oben, von den Männern getrennt. Sie liefen barfuß über die Dächer. Mit Tonkrügen auf dem Kopf gingen sie zum Brunnen. Sie rührten Schafsinnereinen und aufgekochtes Mehl unter das Couscous. Sie beteten auf den Maraboutgräbern. Bei Sonnenuntergang tanzten sie zu den Klängen des Nay auf den Dächern, ihre Bäuche bewegend wie schläfrige Schlangen. Unten mischten die Männer Ziegel an, wickelten Tauchgeschäfte ab, spielten persische Würfen und rauchten die Nargile.

Heute gibt es diese Stadt nicht mehr. Geblieben ist ein Umriss, ein vom Sandriss zerfressenes Heiligtum. Daneben entstand die von Oberst gewünschte neue Stadt. Entworfen von ausländischen Architekten aus dem Ausland. Betonbauten, Antennen. Die Straße wird von großen Bildnissen gesäumt, der Raíz in Wüstengewändern, als Moslem oder als Offizier. Mal ist er gebieterisch und ernst, mal lächelt er mit offenen Armen. Leute sitzen auf leeren Benzinkanistern, knochige Kinder, Greise, die zur Erfrischung an Wurzeln saugen. Lichtleitungen verlaufen schlaff von einem Gebäude zu, anderen. Der glühende Gibli führt Plastiktüten und Müll mit sich, zurückgelassen von Wüstentouristen. Arbeit gibt es keine. Nur gezuckerte Getränke und Ziegen. Und Datteln, die für den Export verpackt werden müssen. Viele junge Menschen gehen fort, zu den Ölfeldern, zu den großen schwarzen Blöcken, zu den ewigen Flammen der Wüste. Es ist eigentlich keine Stadt, es ist eine Ansammlung vieler Leben.

Farid wohnt der Altstadt, in einem der niedrigen Häuser, deren Türen ringsumher auf ein und denselben Hof führen, auf einen verwilderten Garten und eine stets offene Gittertür. Zur Schule geht er zu Fuß. Er rennt mit seinen dünnen Beinen, die sich wie Schilfrohr immerzu schälen. Jamila, seine Mutter, wickelt ihm als Pausenbrot ein paar Sesamstangen in Papier. Wenn er zurückkommt vergnügen sich er und seine Freunde mit einem Blechkarren, der Konservenbüchsen hinter sich herzieht. Oder sie spielen Ball. Farid rollt sich im roten Staub zusammen, wie eine Raupe, stiehlt kleine Bananen und Trauben schwarzer Datteln. Mit einem Seil klettert er ganz nach oben, ins Herz der schattenreichen Pflanzen.

Um den Hals trägt er ein Amulett. Alle Kinder haben eins. Es ist ein kleiner Lederbeutel mit einigen Glasperlen und Fellbüscheln darin. Der böse Blick trifft nur auf das Amulett und du bist in Sicherheit.

Omar, sein Vater, ist Techniker. Er installiert Fernsehantellen. Er wartet auf das Signal, lächelt den Frauen zu, die keine einzige Folge der Ägyptischen TV-Serien verpassen wollen und ihn wie den Retter ihrer Träume behandeln.

Es ist ein Sonnenuntergang ohne Wind. Der Himmel ist pfirsichfarben. Farid setzt sich an die Gartenmauer. Erst jetzt bemerkt er, dass neben ihm ein Tier schnauft. Es steht so dicht bei ihm, dass er sich nicht bewegen kann. Der Schreck springt ihm in die Augen. Er hat Angst, es könnte ein Wadan sein, das Eselsschaf mit den großen Hörnern, von dem viele Sagen berichten. Sein Großvater hatte ihm erzähl, es tauche am Horizont zwischen den Dünen auf, wie eine Fata Morgana. Schon seit vielen Jahren hatte niemand mehr einen Wadan gesehen, doch Großvater Mussa beteuert, es verstecke sich nach wie vor in dem schwarz verkrusteten Wadi, wo sich kein Leben hält und es sei fuchsteufelswild wegen der ganzen Jeeps, die Wüste zerstören, die würden sie mit ihren Rädern verschieben. Doch das Tier hat weder weiße Haarbüschel, noch sichelförmige Hörner. Es fletscht auch nicht die Zähne. Sein Fell ist sandfarben und seine Hörner sind so dünn, dass sie an Strauchzweige erinnern. Farid sieht, dass es eine Gazelle ist, eine junge Gazelle. Sie flieht nicht. Ihre weit aufgerissenen Augen in dieser unmittelbaren Nähe sind klar und ruhig. Durch ihr Fell geht ein Zittern. Vielleicht hat auch sie Angst. Doch auch sie ist zu neugierig auf diese Begegnung, um zurückzuweichen. Langsam streckt ihr Farid einen Zweig entgegen. Die Gazelle öffnet ihr Maul mit den flachen weißen Zähnen und rupft einige frische Pistazien ab. Ohne ihn aus den Augen zu lassen, zieht sie sich zurück. Plötzlich dreht sie sich um, springt über die kleine Lehmmauer und läuft sandaufwirbelnd davon bis hinter den Horizont der Dünen.

Am nächsten Tag in der Schule füllt Farid ganze Seiten mit Gazellen. Er zeichnet sie krumm und schief mit dem Bleistift und er malt sie mit dem Finger aus, den er in mit Wasser verdünnten Temperafarben taucht.

Omar geht seiner Arbeit auf dem Dach nach. Er schließt Stromkabel an und wartet auf den Funken, auf das Zeichen, dass die Fernsehserie gerettet ist. In diesen Tagen kommt der Strom unzuverlässig, stotternd. Das Fernsehen wiederholt regelmäßig einen vom Raiz produzierten Film mit Antony Quinn in der Rolle des legendären Omar al Mukhtar, des Anführers der Beduinen, der wie ein Löwe gegen die italienischen Invasoren kämpfte.

Es ist Nacht, nur ein kleines nacktes Licht brennt. Es zuckt unentwegt, als hätte es Husten. Farid setzt sich an die Gartenmauer. Die Gazelle kommt stets lautlos. Ein leichter Sprung und sie ist da.

Der Umweg by G. Bakker, 2012

An einem frühen Morgen sah sie die Dachse. Sie liefen an dem Steinkreis herum, den sie vor ein paar Tagen entdeckt hatte und gern einmal bei Tagesanbruch sehen wollte. Dachse hatte sie sich immer als friedliche und scheue Wesen vorgestellt, aber hier wurde gekämpft und gefaucht. Als die Tiere sie bemerkten, verschwanden sie ohne Hast zwischen den blühenden Stechginstersträuchern. Sie ging zurück auf dem Weg, den man nur fand, wenn man weit vorausblickte, den sie erahnte, wenn sie auf rostige kissing gates achtete, auf morsche Styles und auf die wenigen Pfähle mit einem Zeichen, das wohl ein gehendes Männchen darstellen sollte. Das Gras war nicht plattgetreten. Sie freute sich über die Dachse. Es tat gut, die Tiere beim Steinkreis zu wissen, auch wenn sie nicht da war.

An dem grasbewachsenen Weg standen uralte Bäume, die Rinde mit hellgrauem Moos bewachsen, die Äste brüchig und doch immer noch belaubt. Die Bäume waren auffallend grün für die Jahreszeit. Der Himmel blieb oft grau. Das Meer war nicht weit entfernt. Wenn sie tagsüber aus einem der Fenster im Obergeschoss schaute, konnte sie es manchmal sehen. An anderen Tagen zeigte es sich nicht. Dann sah sie nur Bäume, hauptsächlich Eichen und hin und wieder hellbraune Kühe, die sie neugierig und zugleich unbeteiligt anblickten. Nachts hörte sie Wasser, ein kleiner Bach führte am Haus vorbei. Ein oder zwei Mal war sie aus dem Schlaf aufgeschreckt, weil der Wind gedreht oder zugenommen und das Wasserrauschen überdeckt hatte. Da war sie etwa drei Wochen hier gewesen. Lange genug, um aufzuwachen, wenn sie ein Geräusch vermisste.

Von den zehn dicken weißen Gänsen auf dem Stück Land neben der Zufahrt waren nach knapp einem Monat noch sieben übrig. Die anderen drei verschwanden bis auf wenige Reste. Verstreute Federn und ein einziges orangefarbenes Bein. Die verbliebenen Tiere grasten ungerührt weiter. Als Räuber konnte sie sich nur einen Fuchs denken. Sie fühlte sich schuldig, weil die Gänse gefressen wurden, glaubte für ihr Überleben verantwortlich zu sein.

Zufahrt war ein großes Wort für den gewundenen Weg von einem oder eineinhalb Kilometern Länge, der nur hier und da behelfsmäßig mit einer Ladung Ziegelsplit befestig war. Das Land beiderseits des Zufahrtsweges, Weiden Sumpf, Wäldchen, gehörte zum Haus. Sie hatte immer noch keine genaue Vorstellung von dem Gelände, weil es so hügelig war. Die Gänseweide war ordentlich mit Stacheldraht umzäunt, immerhin. Er rettete die Tiere nicht. Früher hatte jemand drei kleine Teiche für sie gegraben, die von einer unsichtbaren Quelle gespeist wurden. Früher hatte bei den Teichen auch ein Holzhäuschen gestanden, inzwischen nicht viel mehr als ein zur Seite gekipptes Dach mit einem durchgebogenen Bänkchen davor. Die Zufahrt lag and der Rückseite des Hauses, in der anderen Richtung der Steinkreis und noch ein gutes Stückchen weiter entfernt das Meer. Sehr sanft fiel das Land nach allen Seiten hin ab, sämtliche Fenster boten Aussicht auf tiefer Gelegenes. An der Rückseite waren es nur zwei kleine, eins im großen Schlafzimmer und eins im Bad. Der Bachlauf führte an der Küchenseite vorbei, er folgte dem abfallenden Gelände. Im Wohnzimmer gab es einen großen Holzofen. Die unverblendete Treppe nahm einen Teil der Seitenwand genau gegenüber der Haustür ein. Oben: zwei Schlafzimmer und ein riesiges Bad, darin eine alte Wanne auf Löwenfüßen. Im alten Schweinestall ein größerer Holzvorrat und allerlei Gerümpel. Der Stall hatte einen geräumigen Keller, über dessen Zweck sie sich nicht im Klaren war. Sauber und ordentlich, die Wände glatt mit einer Art Lehm verputzt, ein längliches, niedriges Fenster neben der Betontreppe lies etwas Licht herein. Die Kellerluke konnte mit einer Klappe verschlossen werden, die anscheinend schon lange nicht mehr herunter gelassen worden war.

Erst nach und nach erweiterte sie ihren Raum. Der Steinkreis war kaum mehr als zwei Kilometer entfernt. Einmal war sie zum Einkaufen nach Bangor gefahren. Danach entschied sie sich für Carnarvon, das näher lag. Bangor war eigentlich nicht groß, trotzdem empfand sie den Ort als viel zu laut und voll. Im noch kleineren Carnarvon waren viele Geschäfte geschlossen, auf den Schaufenstern stand in weißer Farbe *For Sale.* Im großen Tesco-Markt war viel Platz und er hatte bis neun Uhr abends geöffnet. Sie schlief im kleineren Schlafzimmer, die Matratze lag auf dem Boden. Wie das große Schlafzimmer, besaß es einen offenen Kamin, aber sie hatte noch kein einziges Mal Feuer darin gemacht.

Der schönste Raum im Obergeschoss war der Flur. Ein X-förmiges Geländer um das Treppenloch, abgenutzte Dielen und eine breite Fensterbank. Auf dieser Fensterbank saß sie manchmal abends und schaute ins Dunkel. Dann sah sie, dass sie nicht ganz allein war. Irgendwo in der Ferne brannte Licht. Anglacy lag in dieser Richtung und von Anglacy gab es Fährverbindungen nach Irland. Einmal hatte sie das Meer im Mondlicht glänzen sehen, eine glatte, bleiche Wasserfläche. Hin und wieder hörte sie von der Gänseweide Geschnatter, gedämpft durch die halbmeter dicken Mauern. Sie war machtlos, sie konnte in der Nacht keinen Fuchs aufhalten. Das große Schlafzimmer hatte sie sich als Arbeitszimmer eingerichtet. Sie hatte den alten Eichentisch, den sie hier vorgefunden hatte, ans Fenster geschoben und eine Schreibtischlampe darauf gestellt. Neben die Lampe kam ein Aschenbecher, neben den Aschenbecher die *Collected Poems* von Amelie Dickinson. Bevor sie sich an den Tisch setzte, öffnete sie meistens das Schiebefenster ein kleines Stück. Wenn sie rauchte, blies sie den Zigarettenrauch in Richtung Spalt. Von hier hatte sie freie Sicht auf die Eichen und Felder. An manchen Tagen aufs Meer und konnte ungestört darüber nachdenken, was so etwas wie Arbeit noch für sie bedeutete. Hinter ihr stand eine Chaiselongue, daneben ein niedriges Tischchen. Sie hatte ein paar Bücher darauf gelegt, las aber keine Zeile. Auf dem Kaminsims stand genau in der Mitte das Portrait von Amelie Dickinson in einem Rahmen.

Die hellbraunen Kühe standen manchmal an der kleinen Bruchsteinmauer, die das umliegende Land von ihrem Grundstück trennte. Sie schienen genau zu wissen, an welchem Fenster sie saß und sie beobachtete. „Mein Grundstück…ich könnte etwas daraus machen“, dachte sie, während sie eine Zigarette nach der anderen rauchte.

Dieses Hügelland voller Bäche und Flüsschen und Baumgruppen war viel zu kompliziert und unübersichtlich für sie. Sie kaufte eine Rosenschere und eine Astsäge in einer Eisenwarenhandlung in Carnarvon. Sie nahm das Haus, wie es war. Ein paar Möbel, ein Kühlschrank und eine Gefriertruhe waren vorhanden. Sie kaufte Teppiche und Kissen, Küchenutensilien, Töpfe, Teller, einen Wasserkessel, Kerzen. Der Holzofen im Wohnzimmer brannte den ganzen Tag. Ein mit Brennöl betriebener Herd heizte die Küche – typisch Britisch. Der riesige Herd diente auch als Durchlauferhitzer für das Wasser. Am Tag ihres Einzugs hatte sie auf dem Küchentisch eine handgeschriebene Bedienungsanleitung gefunden. „Good luck“, wünschte ihr der Verfasser. Sie folgte der Anleitung Schritt für Schritt, wunderte sich kaum, dass sie das Ding tatsächlich in Gang bekam, dass sie am Abend schon die große Wanne mit dampfendem Wasser volllaufen lassen konnte.

Aber die Gänse, über die wunderte sie sich doch ein bisschen. Hatte sie die Vögel mutgemietet? Und eines Morgens graste auf dem Stück Land an der Straße plötzlich eine große Herde schwarzer Schafe. Auf ihrem Grund und Boden! Wem gehörten die Tiere? sie entdeckte, dass der Weg, der zu Steinkreis führte, genau auf die scharfe Biegung ihrer Zufahrt traf. Ein kissing gate in einer Hecke aus gedrungenen Eichen war vollständig von Efeu überwuchert. Durch dieses Weidetor war offensichtlich schon seit Jahren niemand mehr gegangen. Auf der anderen Seite eine Wiese mit hohem braunen Gras. Irgendwo da musste ein Haus stehen. Vom Zufahrtsweg aus sah man in einiger Entfernung einen Hühnerstall, in dem Tag und Nacht ein schwaches Licht brannte. Sie schnitt mit ihrer neuen Rosenschere alle Efeuranken weg. Das Tor ließ sich noch bewegen. Dann fiel ihr auf, dass der Weg über ihr Grundstück verlief, bevor er hinter einem zweiten kissing gate in der Bruchsteinmauer durch die Weiden zu dem hölzernen Steg über den Bach führte. Ein public footpath auf Privatgelände – sie glaubte zu wissen, dass man als Landbesitzer kaum etwas dagegen tun konnte.

Mein Venedig by D. Leon, 2005

Weil es keine Autos gibt, hat Venedig die Freiheit, zumindest für seine Bewohner das zu sein, was es an Zahlen gemessen ist: eine Provinzstadt mit kaum siebzigtausend Seelen, wo eine der wichtigsten Quellen der Unterhaltung der Klatsch ist und wo es folglich keine Geheimnisse gibt. Wenn man irgendetwas über irgendjemanden in Erfahrung bringen will, braucht man sich nur dieser morgendlichen Zufallsbegegnungen zu bedienen und irgendjemand findet sich rasch, der einen vor diesem Antiquitätenhändler, jenem Hautarzt oder einem bestimmten Beamten in irgendeiner Amtsstube warnt. im positiven Sinne kann dieser Zwanglose Meinungsaustausch ebenso gut den ehrlichen Möbelschreiner oder den besten Fischstand auf dem Rialtomarkt zutage fördern. Natürlich kann man sich derartige Informationen überall beschaffen, aber in den meisten anderen Städten erfordert das eine Autofahrt oder einen Telefonanruf. In Venedig läuft man seinen Informationen in die Arme. Und als Schmiergeld ist selten mehr als ein Kaffee und eine Brioche gefordert.

Im Laufe der Jahre gehen Leute dauernd aneinander vorbei. Nach ein paar Monaten tauschen sie ein Nicken, ein Lächeln oder nehmen auf irgendeine andere Weise voneinander Notiz. Obwohl diese Menschen nie aus ihrer freundlichen Anonymität heraustreten, erscheinen sie doch plötzlich mit einem neuen Partner oder mit Kindern, die ihrerseits schon wieder Kinder haben. Sie werden älter, werden langsamer, mitunter verschwinden sie auch ganz von der Bildfläche und immer wieder steht man da und fragt sich, wer sie wohl sind, was sie so tun, wie sie sich in Wirklichkeit verhalten.

Zu den verführerischsten Dingen in Venedig gehört das Gefühl des Geheimnisvollen. Das einem diese Stadt vermittelt. Man weiß nie genau, was einen hinter der nächsten Biegung erwartet oder was hinter einer sich öffnenden Tür zum Vorschein kommt. Schriftsteller, Filmemacher, sogar gewöhnliche Touristen, alle werden gefangen genommen von diesem unheimlichen Gefühl, dass Dinge sich stets als etwas Anderes entpuppen, als was sie auf den ersten Blick zu sein scheinen. Nirgendwo ist das wahrer, als im Fall Alberto Peratonere, dem Custode des Uhrturms von San Marco, Sohn und Enkel von Custodi, Wärtern des Turms. Und nirgendwo zeigt es sich deutlicher, als in der Arbeit, die ihn und sein Vorfahren den Großteil des 20. Jahrhunderts ernährt hat.

Uhr und Turm von San Marco wurden am 1. Februar 1499 eingeweiht und sind seit fünf Jahrhunderten Symbol dieser Stadt. Anders als alle anderen Uhren dieses Alters und dieser Größe, hat diese Uhr zwei Zifferblätter, zwei Gesichter. Das eine blickt an den Statuen von San Teodoro und dem Marcuslöwen vorbei aufs Wasser hinaus, das den ursprünglichen Erbauern der Stadt Sicherheit bot und später die venezianische Flotte zur wirtschaftlichen Eroberung zweier Kontinente trug. Das andere richtet den Blick über die schmale Länge der Merceria nach innen zu Rialto, dem ökonomischen Herzen Venedigs. Wie die Stadt selbst, so ist auch die Uhr gealtert und wurde zweimal, 1757 und 1858 generalüberholt. Luigi Peratonere wurde 1917 zum Custode des Turms und der Uhr von San Marco bestellt. Sein Sohn Giovanni trat 1945 an seien Stelle und Alberto, der jetzige Zustoße übernahm dieses Amt 1986 nach dem plötzlichen Tod seines Vaters. Aufgabe des Uhrwärters ist es, dafür zu sorgen, dass sie geht. Und das heißt, er muss zweimal täglich das große komplizierte Uhrwerk aufziehen und die vielen Einstellungen vornehmen, die nötig sind, damit sie stets die genaue Zeit anzeigt. Nach alter Tradition wohnt der Custode im Turm, was bedeutet, dass er nicht nur gleich neben dem tickenden Herzen der Uhr lebt, sonder auch den atemberaubendsten Blick auf eine Stadt hat, die an sich schon eine endlose Folge von atemberaubenden Anblicken ist.

Custode, der Hüter, der Wärter. In jeder anderen Stadt würde man dabei an einen gebeugten alten Mann mit blauer Schürze denken, dessen Taschen vollgestopft sind mit den sonderbarsten Werkzeugen und der zum Begreifen selbst der einfachsten Dinge etwas mehr Zeit braucht. Aber wir sind in Venedig, wo Weniges so ist, wie es auf den ersten Blick erscheint. Und so hat Alberto Peratonere Philosophie studiert und zu seinem Spezialgebiet Blaise Pascal erwählt. Er ist ein Mann, der durch den Tod seines Vaters mehr oder weniger in diesen Beruf hineingestolpert ist und er zwar das Ticken der Uhr im Blut hat, aber seine wahre intellektuelle Leidenschaft in der Philosophie Pascals findet. Er ist auch keineswegs gebeugt und geschürzt, ein Eigenbrötler oder Hagestolz, sondern ein gut gekleideter, wortgewandter Mann, der auch aus seiner Liebe zu Rita Morosini, seiner Frau, keinerlei Hehl zu machen versucht und seine Leidenschaft für Händels Musik kann er ebenfalls nicht lange verbergen.

Ihn einfach als nichts weiter als den Wärter der berühmtesten Uhr der Welt nach dem Big Ben zu bezeichnen, wäre total falsch. Viel mehr ist er ein Mann, der dadurch, dass er sein Leben lang neben, in gewisser Weise sogar in dem beinah lebenden Mechanismus dieser Uhr zugebracht hat, inzwischen jede ihrer Launen, jedes Rasseln und Quietschen und Klappern kennt. Er weiß Ggenau in welcher Weise sich Luftfeuchtigkeit, Luftdruck und plötzliche Temperaturveränderungen auf das Uhrwerk auswirken und wie man diese mit einem Tropfen Öl bestimmter Dichte oder durch feinfühliges Anpassen eines Hebels wieder ausgleicht. Wenn man ihn fragt, woher er weiß, welches Öl er nehmen, oder wie weit er den Hebel bewegen muss, lächelt Peratonere und antwortet mit Pascals Worten, dass man esprit de finesse benötige, um auf das pulsierende Herz der Uhr eingehen und um ihre vielen Launen verstehen zu können.

Peratonere spricht mit großer Freude darüber, dass Piaget, einer der renommiertesten Uhrenhersteller der Welt großzügig sowohl finanzielle als auch technische Hilfe bei der Restaurierung der Uhr angeboten hat, die in den nächsten zwei Jahren vorgenommen wird. In dieser Zeit soll die Uhr zerlegt und in eine Werkstatt bei Mantua gebracht werden, wo man ausgediente Teile ersetzen wird. Nach ausgiebigen Tests, soll die Uhr dann nach Venedig zurückgebracht und wieder in den Turm eingebaut werden. Am ersten Februar 1999, genau 500 Jahre nach ihrer Einweihung, soll sie wieder in Betrieb genommen werden und für die Venezianer die Minuten und Stunden des Tages zählen. Es ist sehr zu hoffen, dass Alberto Peratonere, Custode und Philosoph, dann auch wieder sein Heim im schlagenden Herzen der Stadt beziehen wird.

Das Casino in der Ca’ Vendramin am Canal Grande, dem Palazzo in dem Richard Wagner 1883 starb, wurde 1945 eröffnet und beschäftig heute knapp 400 Personen, meist Venezianer, davon 280 als Croupiers. In einem durchschnittlichen Jahr macht es 160 Milliarden Lira, von dem die Hälfte direkt an die Commune di Venecia geht und einen Teil jener Mittel ersetzt, die der Stadt durch die Kürzung staatlicher Leistungen entgehen. Über eine halbe Millionen Menschen besuchen jährlich das Casino. Sechshundertdreißigtausend nach dessen eigenen Unterlagen, die meisten sind Italiener und davon die meisten aus dem Venetu.

Das erste, was jedem auffällt, der diesen Palazzo betritt, ist der Blick aus den Vorderfenstern über den Canale Grande hinweg zur Fassade des Palazzo Belloni Battagia. Leider wird dieser Blick von den Gestalten der kettenrauchenden Taxifahrer verdeckt, die hinter den Glastüren des Wassereingangs herumstehen und auf eine Fuhre zum Bahnhof, zum Piazza Roma oder zu irgendeiner Adresse in der Stadt warten. Wenn man in diese riesige hohe Halle tritt, stellt man sich unwillkürlich Sänger mit venezianischen Masken vor, vielleicht eine Gruppe von Vivaldis Weisenmädchen mit einer Kantate, die eigens aus festlichem Anlass komponiert wurde. Stattdessen hört man als erstes das Ping Ping Ping der Münzen, die in den Bäuchen der vielen hundert Spielautomaten an den Wänden einer langen Zimmerflucht zur Linken verschwinden. Manchmal werden die gedämpften Stimmen der Spieler von einem Glockensignal übertönt, das einen neuen Sieg über Göttin Fortuna verkündet.

Meditations

Atembezogen by J. Kabat-Zinn and U. Kesper-Grossman, 2004

Lassen Sie uns diese Sitzmeditation damit beginnen, eine aufrechte und gleichzeitig entspannte Sitzhaltung einzunehmen und ganz besonders den Kontakt des Beckens mit dem Stuhl spüren. Lassen Sie ihr Körpergewicht vom Becken aus zum Boden hin immer mehr los, mit einem Gefühl sich genau hier an diesem Platz, an dem Sie sitzen, ganz nieder zu lassen.

Dann aus dem Becken heraus den Rücken so mühelos wie möglich aufrichten, den Brustkorb weiten und über den Scheitelpunkt sanft nach oben streben. Nehmen Sie Ihre Schultern wahr und lassen Sie sie ganz bewusst nach hinten und unten los.

Nun die Arme und Hände spüren. Wo liegen Ihre Hände jetzt? Vielleicht auf den Knien oder den Oberschenkeln oder locker in Ihrem Schoß. Und nun das Gesicht und auch Ihrer Gesichtsmuskulatur erlauben, weich und gelöst zu sein. Die Augen entweder sanft schließen oder sie leicht geöffnet halten. Und spüren Sie jetzt Ihren ganzen Körper. Vielleicht registrieren Sie Anspannungen im Körper, die im Augenblick unnötig sind und die sich vielleicht jetzt mithilfe der Ausatmung ganz einfach lösen. Lassen Sie Ihre Körperhaltung, so gut es geht, einen Ausdruck einer ganz wachen, aufmerksamen und gleichzeitig ruhigen und empfänglichen inneren Haltung sein.

Atmen Sie, wenn es möglich ist, während der ganzen Meditation durch die Nase und versuchen Sie jedem Augenblick Ihre ganze Aufmerksamkeit zu schenken und die Frage nach der Zeit völlig loszulassen.

Sitzen so still und ruhig wie ein Berg, von Moment zu Moment, Augenblick für Augenblick. Werden Sie sich nun des Ein- und Ausströmen des Atems bewusst. Nehmen Sie genau und unvoreingenommen wahr, wie es sich anfühlt, wenn der Atem über die Nasenöffnungen in den Körper einströmt. Und dann auf die Gleiche Weise alle Empfindungen wahrnehmen, wenn der Atem den Körper über die Nasenöffnungen wieder verlässt. Genau diese Einatmung spüren und genau diese Ausatmung. Genau hier und genau jetzt. Sie können den Atem in den Nasenöffnungen beobachten oder Sie betrachten das sanfte Heben und Senken derBauchdecke oder die Empfindungen des Ein-und Ausatmens im Brustkorb. Am besten ist es, wenn Sie die Aufmerksamkeit dort ruhen lassen, wo Sie den Atem jetzt am

deutlichsten im Körper wahrnehmen und wo die Empfindungen für Sie am angenehmsten

sind. Vollkommen wach und präsent für die gesamte Dauer der Einatmung und wach und präsent für die gesamte Länge der Ausatmung. Und auch die kurze Atempause zwischen Aus- und Einatmung bewusst wahrnehmen. Lassen Sie so gut Sie können, alle Vorstellungen und Konzepte darüber los, wie der Atem jetzt sein sollte. Erlauben Sie ihm, in seinem eigenen, ganz natürlichen Rhythmus zu kommen und zu gehen, ihn weder beschleunigen, noch versuchen, ihn zu vertiefen und verlangsamen. Jede Einatmung empfangen und ausatmend den Atem in seiner eigenen

Zeit ausströmen lassen. Ganz wach von Moment zu Moment, von Atemzug zu Atemzug.

Erlauben Sie sich, hier zu sitzen und von Moment zu Moment mit allen Empfindungen im

Körper zu atmen. Schauen Sie, ob Sie alle Körperempfindungen, die angenehmen ebenso

wie die unangenehmen, in einem weiten, offenen und annehmenden Gewahrsein halten

können. Nehmen Sie Ihren ganzen Körper wahr wenn Sie einatmen und wenn Sie ausatmen,

so als würde der Atem über die Poren der Haut ein- und wieder ausströmen. Sehen, wie alle

Empfindungen entstehen und sich wieder auflösen und wie der Atem kommt und geht. Und nun für die verbleibende Zeit in Stille und Achtsamkeit verweilen, vollkommen gegenwärtig und annehmend für die sich vom Moment zu Moment entfaltende Erfahrung. Nichts hinzufügen und nichts ausschließen. Genau jetzt, von Augenblick zu Augenblick, von Atemzug zu Atemzug.

Körperbezogen J. Kabat-Zinn and U. Kesper-Grossman, 2004

Nehmen Sie eine Haltung ein, die sich für Sie im Moment am passendsten anfühlt. Und lassen Sie sich ein auf die Wahrnehmung des Atems, wie er in Ihrem Körper ein und ausströmt. Während Sie hier sitzen oder liegen.

Gehen Sie in Kontakt mit den Empfindungen des Atmens, überall dort, wo diese am lebendigsten für Sie sind. Vielleicht an den Nasenflügeln. Oder im Bauchbereich oder auch im Brustraum und reiten Sie Moment für Moment auf den Wellen Ihres Atems.

Erleben Sie die gesamte Dauer des Einatmens und die Zeit die der Atem braucht, um den Körper wieder zu verlassen.

Richten Sie die Aufmerksamkeit auf diese Atemempfindungen und halten Sie sie dort, so gut Sie können. Verweilen Sie in diesem Gewahrsein des Atems. Augenblick, für Augenblick und Atemzug für Atemzug. Und wenn Sie möchten können Sie nun den Fokus Ihrer Aufmerksamkeit auf den Kontakt des Körpers mit dem Stuhl oder dem Boden richten und die damit verbundenen Empfindungen spüren. Erlauben Sie diesen Empfindungen genauso zu sein, wie sie sind. Ganz egal ob Sie sie eher als angenehm oder unangenehm oder neutral empfinden. Bleiben Sie einfach bewusst bei diesen Empfindungen in einem Gewahrsein, das gleichzeitig auch den Atem umfasst bemerken Sie einfach, wie der Atem alle Empfindungen von Kontakt, von Berührung von Temperatur und Druck, Härte und Weichheit mit umfassen kann. Wie der Atem diese Empfindungen gleichsam in Aufmerksamkeit badet.

Nehmen Sie die Empfindungen genauso wahr, wie sie sind außerhalb des Denkens, nur als reine Empfindungen ob sie schwach sind, mittel, oder stark in ihrer Intensität ob angenehm, unangenehm oder neutral. Sie brauchen nichts an ihnen zu verändern. Öffnen Sie sich ihnen einfach nur mit Bewusstheit während Sie im gegenwärtigen Moment ruhen, so wie er ist, Augenblick für Augenblick für Augenblick.

Und wenn Sie bereit sind, erlauben Sie sich jetzt das Feld ihres Gewahrseins noch weiter auszudehnen, so dass es die ganze Haut und die Luft um den Körper herum mit einschließt und die Wahrnehmung des Körpers als Ganzes. wie er hier sitzt oder liegt und atmet.

Sodass Ihr Gewahrsein dieses ganze Universum von Empfindungen einschließt, das man den Körper nennt. oder das man als die Erfahrungslandschaft des Körpers bezeichnen könnte… vielleicht nehmen sie weiterhin auch noch die Empfindungen des Atems wahr, der ein und ausströmt aber jetzt im größeren Kontext des gesamten Körpers, der atmend hier sitzt oder liegt.

Während Sie einfach nur hier verweilen, im Bewusstsein des Körpers als Ganzes in seiner Vollständigkeit. In seinem ganz eigenen Dasein. Umfasst Ihr Gewahrsein den Körper. Erfüllt den Körper. Umgibt ihn. Fließt durch ihn hindurch, überall. So wie auch der Atem überall hinfließt. Vielleicht können Sie sogar spüren oder sich vorstellen, wie Ihre Haut atmet, was sie in der Tat auch tut.

Body-scan by J. Kabat-Zinn, 2007

Wenn wir die Sitzmeditation praktizieren, ist es hilfreich eine Körperhaltung einzunehmen, die einem inneren Gefühl der Würde entspricht. Der Rücken ist, so gut es geht, aus dem Becken heraus aufgerichtet. Die Schultern sinken nach hinten und der Hinterkopf strebt leicht nach hinten und oben.

Die Hände liegen entweder im Kontakt miteinander in Ihrem Schoß oder ruhen einspannt auf Ihren Knien oder Oberschenkeln. Die Augenlider nun sanft schließen, wenn dies angenehm für Sie ist, und wenn nicht, dann halten Sie die Augen einfach leicht geöffnet. und schauen Sie mit entspanntem Blick auf den Boden oder auf die Wand vor Ihnen. Und nun, wenn Sie so weit sind, dann bringen Sie Ihre Aufmerksamkeit sanft zu den Atemempfindungen an der Bauchdecke und spüren Sie das sanfte Heben und Ausdehnen der Bauchdecke mit jeder Einatmung und das Senken und leichte Zusammenziehen mit jeder Ausatmung. Atemzug für Atemzug das Heben bei der Einatmung spüren und das Senken bei der Ausatmung. Genau diese Einatmung und genau diese Ausatmung. Von Moment zu Moment nur bewusst wahrnehmen, was Ihnen durch den Kopf geht, was in Ihrem Körper geschieht und was Sie fühlen.

Ganz aufmerksam für den Körper und für den Atem in diesem Augenblick und in diesem… und diesem. Wenn Sie möchten, dann durchwandern Sie jetzt gleich den ganzen Körper mit Ihrer Aufmerksamkeit. Von den Fußsohlen bis hinauf zum Scheitelpunkt. Lassen Sie dabei Nacheinanderjede einzelne Körperregion ins Bewusstsein treten und spüren Sie welche Empfindungen gerade da sind. Vielleicht nehmen Sie den Atem wahr oder Sie registrieren die

Abwesenheit jeglicher Empfindungen in einer bestimmten Körperregion, wenn das so sein sollte. Und während Sie dann mit Ihrer Aufmerksamkeit den ganzen Körper nach und nach von innen erspüren, wird vielleicht gleichzeitig das Gewahrsein immer offener und immer feiner. Beginnen wir also nun damit, die Aufmerksamkeit auf beide Füße zu richten. Beide Füße gleichzeitig. Dann die Fußgelenke. Unterschenkel. Die Knie. Und jetzt die Oberschenkel. Die ganze Beckenregion. Der untere Rücken. Und der Bauch. Die Magengegend direkt unterhalb des Brustkorbs. Die mittlere Rückenpartie. Und der obere Rücken mit den Schulterblättern und dem Bereich zwischen den Schulterblättern. Der ganze Brustkorb und besonders die Brustmitte. Die Schultern und dann, wenn Sie soweit sind, zu

den Händen hinuntergleiten, beide Daumen spüren, alle Finger, die ganzen Hände. Handgelenke und Arme, der Hals und Nacken. Der Hinterkopf und die Schädeldecke. Und dann Ihr Gesicht. Die Augen in den Augenhöhlen. Die Lippen, der Mundraum und die Zunge im Mund und dann der ganze Kopf. Und nun den ganzen Körper spüren. Und allmählich den Fokus auf den Körper wieder loslassen.

Wahrnehmen, wie der Atem kommt und geht und auch hier die Aufmerksamkeit jetzt nicht mehr auf einen bestimmten Ort im Körper fixieren. Allmählich nun die Aufmerksamkeit wieder auf den Körper richten und ihn in seinen vertrauten grenzen wahrnehmen.

Wenn sich nun möglicherweise die Grenzen zwischen dem, was wir innen, und dem, was wir

außen nennen, beginnen immer mehr aufzulösen, entsteht vielleicht Raum für ein Gefühl der Stille. Ein Gefühl des Ganzseins und der Verbundenheit.

Translations of the interventions into English language

Stories

The Sea in the Morning by M. Mazzantini, 2011

Farid has never seen the sea, never diven into it. He often imagined it – covered with stars like a pasha’s coat, blue, like the blue wall of the dead city. He looked for the fossilized shells that had been buried millions of years ago when the sea flooded the desert, traced the lizard fish that swim under the sand, saw the salt lake and the Bitter Lake and the silvery dromedaries that move like battered pirate ships.

He lives in one of the last oases in the Sahara. His ancestors belonged to a tribe of nomadic Bedouins. They set up their tents in *kadis* – dried river beds covered with plants. The goats grazed. The wives prepared meals on glowing-hot stones. They had never left the desert. A certain distrust of the people on the coast prevailed. Traders, pirates.

The desert was their home, wide and boundless. Their sand sea. Spotted with dunes, like a jaguar’s fur. They owned nothing. Nothing but footprints they laid in the sand. The sun moved the shadows. They were used to defying thirst and drying up like dates, without dying. A dromedary blazed their trail, a long, slanted shadow. They disappeared into the dunes. We are invisible to the world, but not to God. They travelled around with this thought in their hearts. In the winter, the north wind that came from the rocky ocean made the woolen clothes on their bodies stiff and their skins bloodless, like the goatskins on their drums, clutched to their bones. Ancient witchcraft fell from heaven. The sand troughs were sharp as knives. Touching the desert meant injury. The old people were buried where they died, left to the silence of the sand. The Bedouins moved on. Rags in white and indigo.

New dunes arose in the spring, rosy and pale. Sand virgins.The blazing Gibli came with the raspy moans of the jackal. Little twisters nipped the sand here and there like travelling ghosts, low-flying gusts, sharp as scimitars. An aroused army. The desert arose in a second and swallowed the sky. There was no more threshold to the hereafter. The Bedouins bent under the burden of the grey storm, sought protection behind the bodies of crouching animals like under the cloak of an old curse.

They had stayed there. They had made a wall of clay and fenced in pasture. There were wheel tracks in the sand. Sometimes a caravan passed through. They were on the route of traders’ from Black Africa who shortened their path to the sea through the desert. They had ivory, incense, precious stones, and captive people, who were to be sold as slaves in the harbors of Cyrenaica and Tripolitania. The traders took nourishment in the oasis. They ate, drank. A city had arisen. Walls of dried clay, resembling braided ropes. Rooves of palm fronds. The women lived above, separated from the men. They ran barefooted over the roofs. They went to the wells with clay jugs on their heads. They stirred sheep innards and boiled flour into the couscous. They prayed on the graves of marabouts. At dusk they danced on the roofs to the sound of the nay, moving their bellies like sleepy snakes. Below the men mixed clay bricks, made trades, played Persian dice, and smoked water pipes.

This city no longer exists. An outline remains, a sanctuary eroded by sand. Only an outline remains, a sanctuary eroded by sand. The colonel’s new city arose next to it. Designed by foreign architects. Concrete buildings, antennas. The street is lined with big portraits, the Raiz wearing desert clothing, dressed as a Moslem or an officer. Sometimes imperious and ernest, sometimes smiling with open arms. People are sitting on empty gas cans, boney children, old men sucking on roots for refreshment. Power lines hang loosely from one building to the next. The blazing Gibli carries plastic bags and garbage with it, left by desert tourists. There is no work. Only sweetened drinks and goats. And dates that have to be packed for export. Many young people leave for the oil fields, to the big black blocks, to the eternal flames of the desert. It’s not really a city, it’s a collection of many lives.

Farid lives in the old city, in one of the low houses whose doors form a circle around a single courtyard, an unkept garden and an iron gate that is always open. He goes to school on foot. He runs on his thin legs that peel like reeds. Jamila, his mother, wraps a few sesame sticks in paper for his snack. When he returns, he and his friends play with a tin wagon that pulls tin cans behind it. Or they play ball. Farid curls up in the red dust like a caterpillar, steals little bananas and clusters of black dates. He climbs to the top with a rope, into the heart of the shadowy plants.

He wears an amulet around his neck. All the children have one. It is a little leather pouch containing some glass beads and tufts of fur. The evil eye only falls on the amulet, and you are safe.

Omar, his father, is a repairman. He installs TV components. He waits for the signal, smiles at the women, who don’t want to miss a single episode of the Egyptian TV series and treat him like the rescuer of their dreams.

It is a sunset without wind. The sky is peach pink. Farad sits on the garden wall. He suddenly notices that an animal is breathing next to him. It is standing so close to him that he cannot move. Fear springs into his eyes. He’s afraid it could be a *wadan*, the donkey-sheep with the big horns described in many sagas. His grandfather told him that it appears on the horizon between the dunes like a Fata Morgana. No one has seen a *wadan* for many years, but grandfather Mussa claims it still hides in the black, caked wadi, where there is no life, and it is furious because of the many jeeps which destroy the desert, which displace it with their wheels. But the animal has neither white tufts of hair nor sickle-shaped horns. It doesn’t bare its teeth. Its fur is sand-colored, and its horns are so thin that they resemble the twigs of a bush. Farid sees that it is a gazelle, a young gazelle. It doesn’t run away. Its wide-open eyes right next to him are clear and calm. But its coat is shivering. Maybe it is also afraid. It is too curious to back off. Farid slowly offers it a branch. The gazelle opens its mouth with flat, white teeth and tears off a few fresh pistachios. Without letting him out of its sight, the gazelle retreats. It suddenly turns around, leaps over a little adobe wall and runs off in a whirl of sand to behind the skyline of the dunes.

On the next day in school, Farid fills entire pages with gazelles. He draws the crooked and slanted with a pencil, or he paints them with a finger that he dips in diluted tempera watercolor.

Omar does his work on the roof. He connects electric cables and waits for the sparks, for the sign that the TV series is rescued. Electricity is unreliable in these days and only comes in sputters. The television repeatedly shows a film produced by the Raiz with Anthony Quinn in the role of the legendary Omar al Mukhtar, the leader of the Bedouins, who fights like a lion against the Italian invaders.

It is night; only a small, naked light burns. It flickers continually, as if it had a cough. Farid sits on the garden wall. The gazelle always comes soundlessly. A little leap, and it is there.

The Detour by G. Bakker, 2012

She saw the badgers one early morning. They were running around the stone circle that she had discovered a few days earlier and wanted to see at daybreak. She had always imagined badgers as peaceful and clever creatures, but here they were fighting and hissing. When the animals noticed her, they disappeared unhurriedly between the blooming gorse bushes. She returned on the path that only could be found if one looked far ahead. She sensed it when she looked at the rusty kissing gates, at decaying styles, and at the few posts with a sign, that probably was meant to depict a little walking man. The grass was not trodden down. She was happy about the badgers. It was good to know about them at the stone circle, even if they weren’t there.

Ancient trees, their bark covered with light-green moss and their brittle branches still bearing leaves, stood along the grassy path. The trees were strikingly green for the time of year. The sky was often grey. The ocean was not far away. When she looked out the window in the upper story, she could sometimes see it. On other days, it remained invisible. Then she only saw trees, mostly oaks, and now and then light-brown cows that looked at her curiousily, but, at the same time, uninvolved. She heard water at night, a little stream flowing past the house. She awoke startled once or twice because the wind had changed direction or increased and smothered the sound of water rushing past. She had been here for about three weeks. Long enough to wake up when she didn’t hear a familiar sound.

Of the ten fat white geese on the piece of land next to the driveway, only seven remained after barely a month. The other three had disappeared except for a few remains. Scattered feathers and a single orange-colored leg. The rest of the animals just continued grazing. She could only imagine a fox as the thief. She felt guilty because the geese had been eaten and believed she was responsible for their survival.

Driveway was a big word for the winding lane, about one or one and a half kilometers long, that was only provisionally paved with a load of crushed tile here and there. The land on either side of this lane consisting of a reedy marsh and a little forest belonged to the house. She still had no exact notion of the terrain because it was so hilly. At least the goose meadow was orderly and surrounded by barbed wire. It didn’t save the animals. Someone had dug three little ponds that were fed by an invisible spring for them at some earlier time. There had also been a little wooden shelter next to the pond, but now there was little more than a roof toppled to one side with a little, sagging bank in front of it. The driveway was at the back of the house, in the direction opposite the stone circle and quite a bit further from the ocean. The terrain sloped gently on all sides; low-lying ground could be seen from all windows. There were two small ones at the back of the house, one in the large bedroom, and one in the bathroom. The stream ran past the kitchen, following the sloping terrain. There was a large wood stove in the living-room. The naked stairway occupied part of the wall directly across from the front door. Above: two bedrooms and a huge bathroom with an old bathtub standing on lions’ feet. There was a big supply of firewood and all kinds of junk in the pigsty. The stall had a roomy cellar, but she had no idea what it was used for. Clean and orderly, the walls plastered with some kind of adobe, a long, low window next to the concrete stairs let in some light. The cellar skylight could be closed with a hatch, which apparently had not been lowered for a long time.

She gradually expanded her space. The stone circle was hardly more than two kilometers away. Once she had gone to Bangor to shop. Afterwards she decided on Carnavon, which was closer. Bangor wasn’t really big, but she still found the place to be too loud and crowded. In smaller Carnavon many shops were closed; *For Sale* was written in white on the shop windows. There was a lot of room in the big Tesco supermarket, and it was open until 9 PM.

She slept in the little bedroom; the mattress lay on the floor. Like the big bedroom, it had a fireplace, but she had never lit a fire in it.

The most beautiful room in the upper story was the hall. An x-shaped banister around the stairway opening, worn floorboards and a broad window bank. Sometime she sat on the window bank in the evening and looked out into the dark. Then she saw that she wasn’t alone. Somewhere far away a light was burning. Anglacy lay in that direction, and there were ferries from Anglacy to Ireland. Once she saw the ocean shining in the moonlight, a smooth, pale expanse of water. Now and again she heard the geese chattering in their meadow, muted through the half-meter-thick walls. She was powerless; she couldn’t stop a fox in the night.

She had furnished the large bedroom as a workroom. She pushed the old oak table she had found to the window and placed a desk lamp on it. An ashtray next to the lamp, and the *Collected Poems* of Amelie Dickenson next to it. Before she sat down at the table, she usually opened the sliding window a little. When she smoked, she blew the cigarette smoke in the direction of the opening. She could clearly see the oaks and fields from here. She could see the ocean on some days and could peacefully think about it, which meant something like work to her. A chaise longue stood behind her and next to it, a low little table. She had laid a few books on it, but hadn’t read a line. There was a portrait of Amelie Dickinson in a frame right in the middle of the fireplace mantle.

The light-brown cows sometimes stood beside the little rubble-stone wall which separated the surrounding land from her property. They seemed to know exactly which window she was sitting at and watched her. “My property…I could make something of it,” she thought while she smoked one cigarette after another.

The hilly terrain full of streams and little rivers and groups of trees was much too complicated and confusing for her. She bought a rose clipper and a pruning saw in a hardware shop in Carnarvon. She accepted the house as it was. A few pieces of furniture, a refrigerator, and a freezer were there. She bought rugs and pillows, kitchen utensils, pots, plates, a water-cooker, candles. The wood stove in the living room burned the whole day. An oil-burning stove heated the kitchen - typically British. The huge stove also served as a flow heater for the water. She had found hand-written instructions for use on the kitchen table on the day she moved in. The author wished her “Good luck”. She followed the instructions step by step and was hardly surprised that she actually got it to work, that she could fill the big bathtub with steaming water that evening.

But the geese, she was a little surprised about them. Had she also rented them? Suddenly one morning a large herd of black sheep was grazing on the piece of land next to the driveway. On her piece of land! Who owned the animals? She discovered that the path to the stone circle joined her driveway exactly at the sharp curve. A kissing gate in a hedge of squat oaks was completely covered with ivy. Apparently no one had gone through this pasture gate for years. There was a meadow with high brown grass on the other side. A house must stand somewhere there. From the driveway one could see in the distance a chicken coop in which a weak light burned day and night. She cut all the ivy away with her new rose clippers. The gate could still be moved. Then she noticed that the path crossed her property before it continued behind a second kissing gate in the stone-rubble wall through the reeds and led to the little wooden bridge over the stream. A public footpath on private property – she believed that a landowner could hardly do anything about it.

My Venice by D. Leon, 2005

Because there are no cars, Venice has the freedom, at least for its residents, to be what it is according to numbers: a provincial city with hardly 70,000 inhabitants, where gossip is one of the most important sources of entertainment and where, as a result, there are no secrets. If you want to find out something about someone, you only need to take part in a chance morning meeting, and someone is quickly found who warns you about this antique dealer, that family doctor, or a certain civil servant in some office. In a positive sense, this casual exchange of opinions can just as well reveal the honest carpenter or the best fish stand at the Rialto market. You can, of course, get this kind of information anywhere, but this requires a telephone call or driving somewhere in most other cities. In Venice you run into the open arms of this information. And rarely more than a coffee and a brioche are demanded as bribery.

Over the years, people walk past one another. After a few months, they exchange a nod, a smile or take notice of one another is some other way. Although these people never step out of their friendly anonymity, they suddenly appear with a new partner or with children that have children of their own. They age, slow down, they even disappear from the scene entirely, and one often stands there and wonders who they are, what they do, how they really behave.

One of the most captivating things in Venice is the feeling of mystique the city conveys. You never know exactly that to expect behind the next turn or what will appear behind an opening door. Writers, filmmakers, even common tourists, all are captured by this uncanny feeling that things continually turn out to be something else that what they appeared to be at the first glance. Nowhere else is this truer than the case of Alberto Peratonere, the custodian of the clock tower of San Marco, son and grandson of custodians, guardians of the tower. And nowhere is this shown more vividly than in the work that fed has him and his ancestors for greater part of the 20^th^ century.

The clock and the tower of San Marco were inaugurated on February 1, 1499, and have symbolized this city for five centuries. In contrast to all other clocks of this age and this size, this one has two dials, two faces. One looks past the statues of San Teodoro and the Marcus lions over the water which offered the original builders of the city safety and later carried the Venetian fleet to the commercial conquest of two continents. The other directs its gaze over the narrow length of the Merceria inside to the Rialto, the economic heart of Venice. Like the city itself, the clock has aged and was rebuilt twice, in 1757 and 1858. Luigi Peratonere was appointed custodian of the tower and the clock of San Marco in 1917. His son Giovanni replaced him in 1945, and Alberto took over the post in 1986 after the sudden death of his father. The clock custodian’s duty is to keep it running. That means he must wind up the big, complicated clockwork twice daily and perform the many adjustments that are necessary so that it always shows the exact time. The custodian lives in the tower according to an old tradition, which means that he not only lives right next to the ticking heart of the clock, but also has a breath-taking view of the city, which itself consists of an endless series of breath-taking views. Custodian, guardian, caretaker, in any other city you would picture a bent old man in a blue apron whose pockets are stuffed full of the most peculiar tools and who needs somewhat more time to understand even the simplest things. But we’re in Venice, where few things are what they appear to be at first glance. Alberto Pertonere studied philosophy and chose Blaise Pascal as his major. He is a man who more of less stumbled into this profession after the death of his father and has the ticking of the clock in his blood, but his real intellectual passion is Pascal’s philosophy. He is also in no way bent and wearing an apron, a loner or confirmed bachelor, but a well-dressed, eloquent man who does not try to conceal his love for his wife, Rita Morosini, or his passion for Handel’s music.

It would be entirely wrong to simply call him the guardian of the most famous clock in the world after Big Ben. He is rather a man who, having spent his life long near or, in a certain sense, in the almost living mechanism of this clock, in the meantime knows each of its moods, every rattle, squeak, and clatter. He knows exactly how humidity, air pressure, and sudden changes in temperature affect the clockwork and how to compensate this with a drop of oil with a certain density or by delicately adjusting a lever. If you ask him how he knows which oil to use or how far the lever must be moved, Peratonere smiles and answers with Pascal’s words, one needs ‘esprit de finesse’ to cater to the pulsing heart of the clock and to be able to understand its many moods.

With great pleasure Peratonere recounts that Piaget, one of the most renowned clockmakers in the world, offered generous financial and technical help in restoring the clock, which will be performed in the next two years. At this time, the clock will be taken apart and taken to a workshop in Mantua, where worn-out parts will be replaced. After extensive tests, the clock will be returned to Venice and remounted in the tower. On the first of February, 1999, exactly 500 years after its inauguration, it is to be put back into operation and to count the minutes and hours of the day for the Venetians. We can only hope that Alberto Peratonere, custodian and philosopher, will move back into his home in the beating heart of the city.

The casino in the Ca’ Vendramin on the Canale Grande, the palace in which Richard Wagner died in 1883, was opened in 1945 and employs just under 400 people, mostly Venetians, 280 of whom are croupiers. In an average year it earns 160 billion lira, half of which goes directly to the Commune di Venecia and replaces part of the money that the city lacks due to a cutback in public services. Over half a million people visit the casino every year. 630,000 according to their records, most of whom are Italians, and most of them from Venetu.

The first thing you notice when you enter this palace is the view over the Canale Grande to the façade of the Palazzo Belloni from the front windows. Unfortunately, this view is obscured by the figures of chain-smoking taxi drivers standing around the glass doors of the water entrance and waiting for a passenger to the train station, the Piazza Roma, or some address in the city. When you enter this huge, high hall, you automatically imagine singers with Venetian masks, perhaps a group of Vivaldi’s orphan girls with a cantata which was composed especially for a festive event. Instead, the first thing you hear is the ping-ping of coins disappearing into the bellies of the many hundred slot machines on the walls of a long suite of rooms on the left. Sometimes the muted voices of gamblers are drowned out by the bell that announces a new victory over the goddess Fortuna.

Meditations

Breathing exercises by J. Kabat-Zinn and U. Kesper-Grossman, 2004

Let us begin this sitting meditation by taking an upright, but relaxed, sitting position; be especially aware of the contact of your pelvic bones with the chair. Release your body weight from your pelvis to the ground more and more with the feeling of settling down exactly here in this place on which you are sitting. Then straighten your back as easily as possible from your pelvis, expand your chest and gently aspire to rise from the top of your head. Be aware of your shoulders and consciously let them fall backwards and downwards.

You only feel your arms and hands. Where are your hands right now? Maybe on your knees or thighs or resting loosely in your lap. And now let your face and your facial muscles become soft and relaxed. Either gently close your eyes or keep them slightly open. Now become aware of your entire body. Perhaps you notice tensions in your body which are unnecessary right now and can perhaps be easily released by breathing. Let you position, as much as possible, become an expression of an entirely awake, attentive and, simultaneously calm and receptive inner mindset.

Breathe, if possible, through your nose during the entire meditation and try to direct your full attention to your breathing at each instant and completely let go of the question about the time.

Sit as still and calm as a mountain, from moment to moment. Instant to instant. Become conscious of your breath flowing in and out. Open up your mind to how it feels when air streams through your nostrils into your body. And then, in the same way, perceive all the sensations when the air leaves your body through your nostrils. Concentrate just on this breathing in and out. Just the here and now. You can contemplate the breath in your nostrils or observe the gentle swelling of your breast and sinking of your belly or the impressions of breathing in and out in your chest. It is best to let your attention rest where you most intensively perceive breathing in your body and where these feelings are most pleasant. Completely awake and in the present for the entire time you breathe in and awake and in the present for the entire time you breathe out. And also be completely aware of the short pause between breathing in and breathing out. Release, as well as you can, all ideas and concepts of how your breathing should be now. Allow it to come and go in its own natural rhythm; don’t try to speed it up or to breathe deeper or slower. Welcome each inspiration and let your breath out in its own time. Completely awake from moment to moment, from breath to breath. Allow yourself to sit here and to breathe with all the sensations in your body, from moment to moment. See if you can keep all your body sensations, the pleasant ones as well as the unpleasant ones, in a wide, open, and accepting awareness. Be aware of your entire body when you breathe in and when you breathe out as if the breath were flowing in and out again through your pores. Notice how all sensations arise and disperse and how your breath comes and goes. Remain in this tranquility and awareness for the rest of the time. Do not add anything and do not exclude anything; stay completely in the present and accept the unfolding experience from moment to moment. Do not add anything and do not exclude anything. Just now, from moment to moment, from breath to breath.

Body-Related by J. Kabat-Zinn and U. Kesper-Grossman, 2004

Assume a position that feels best for you at the moment. Accept the perception of your breathing, how it flows in and out of your body while you are sitting or lying here.

Get into contact with the sensations of your breathing, everywhere where it feels liveliest to you. Maybe in your nostrils. Or in your belly or chest and ride, moment by moment, on the wave of your breath. Experience the entire length of breathing in and the time your breath needs to leave your body.

Direct your attention to these breathing sensations and maintain it there as well as you can. Dwell on this conscious breathing. Moment for moment and breath by breath. If you want to, you can now focus your attention on the contact of your body with the chair or the floor and feel the sensations that arise. Let these sensations be just as they are. It doesn’t matter if you find them pleasant or unpleasant or neutral. Simply remain aware of these sensations, which also include your breathing, and notice how your breathing includes all sensations of contact, touch, temperature and pressure, hardness and softness. How your breath bathes these sensations in awareness.

Perceive these sensations just like they are, outside of thought, only as pure sensations, whether weak, moderate, or strong in their intensity, whether pleasant or unpleasant or neutral. You needn’t change anything about them. Simply open up to them consciously while you rest in the present moment, just as it is, moment by moment by moment.

And when you’re ready, let the field of your consciousness widen even further so that it encompasses all your skin and the air around your body and the awareness of your body as a whole, how it is sitting or lying here and breathing. So that the awareness includes this entire universe of sensations which we call our body or that one could describe as the body’s landscape of experience. Perhaps you are also aware of your breathing sensations, air flowing in and out, but now in the context of your entire body that is sitting or lying here breathing.

While you just rest here, conscious of your body as a whole in its completeness, with its very own existence. Your awareness encloses your body. Satisfies your body. Surrounds it. Flows through it, everywhere. Just like your breath is flowing everywhere. Perhaps you can even feel or imagine how your skin breathes, which it really does.

Body-scan by J. Kabat-Zinn, 2007

When we perform sitting meditation, it helps to take a position which reflects an inner feeling of dignity. You straighten your back is, as much as possible, right up from your pelvis. Your shoulders fall backwards, and the back of your head is tilted back and upwards.

Your hands are laying either in contact with each other in your lap or rest on your knees or thighs. Close your eyes softly if this is pleasant for you, and if not, then keep them slightly open and direct a relaxed gaze to the floor or the wall in front of you. And when you are ready, direct your attention gently to the breathing sensations in your belly and feel the gentle lifting and expanding when you breathe in and the sinking and slight contraction when you breathe out. Breath by breath, feel the rise when you breathe in and the fall when you breathe out. Just this breathing in and out. From moment to moment in conscious awareness, whatever goes through your mind, what is happening inside your body, and what you feel.

Completely attentive to your body and breathing in this moment and in this…and this…. If you wish, wander through your entire body with your awareness. From the soles of your feet to the top of your head. Let each single body region enter your consciousness, one after another and feel what sensations are there right now. Perhaps you perceive your breathing or you register the absence of any sensations in a certain body region if that is the case. And while you sense your entire inner body bit by bit, your awareness may increasingly become more open and finer. Let us begin by directing our attention to both feet. Both feet at the same time. Then the ankles and the lower legs. The knees. And now the thighs. The entire pelvis. The lower back. And the belly. The stomach directly under the ribcage. The middle of your back. The upper back and the shoulder blades and the area between the shoulder blades. The entire thorax and especially the middle of your chest. The shoulders and then, when you are ready, glide down to your hands; feel both thumbs, all your fingers, your entire hands. Wrists and arms, throat and neck. The back of your head and the top of your head. And then your face. Your eyes and eye sockets. Your lips, your mouth, and the tongue in your mouth, and your whole head. And now feel your entire body. Slowly release the focus of your attention from your body.

Perceive how your breath comes and goes and here also, don’t fix your attention on a certain place in your body. Gradually turn your attention to your body again and perceive it in its familiar boundaries.

When the possible boundaries between what we call inside and what we call outside begin to dissolve, they may arise room for a feeling of peace. A feeling of entirety and solidarity.
